# Just give the contrast? Appraisal of guidelines on intravenous iodinated contrast media use in patients with kidney disease

**DOI:** 10.1186/s13244-024-01644-5

**Published:** 2024-03-18

**Authors:** Jingyu Zhong, Liwei Chen, Yue Xing, Junjie Lu, Yuping Shi, Yibin Wang, Yi Deng, Run Jiang, Wenjie Lu, Silian Wang, Yangfan Hu, Xiang Ge, Defang Ding, Huan Zhang, Ying Zhu, Weiwu Yao

**Affiliations:** 1 Department of Imaging, Tongren Hospital, Shanghai Jiao Tong University School of Medicine, Shanghai, 200336 China; 2 Department of Epidemiology and Population Health, Stanford University School of Medicine, Stanford, CA 94305 USA; 3 Department of Nephrology, Tongren Hospital, Shanghai Jiao Tong University School of Medicine, Shanghai, 200336 China; 4 Department of Urology, Tongren Hospital, Shanghai Jiao Tong University School of Medicine, Shanghai, 200336 China; 5 University of Washington School of Pharmacy, Seattle, WA 98105 USA; 6 Department of Pharmacovigilance, Shanghai Hansoh BioMedical Co., Ltd, Shanghai, 201203 China; 7 Department of Radiology, Ruijin Hospital, Shanghai Jiao Tong University of Medicine, Shanghai, 200025 China

**Keywords:** Acute kidney injury, Contrast media, Glomerular filtration rate, Practice guideline

## Abstract

**Objective:**

To appraise the quality of guidelines on intravenous iodinated contrast media (ICM) use in patients with kidney disease, and to compare the recommendations among them.

**Methods:**

We searched four literature databases, eight guideline libraries, and ten homepages of radiological societies to identify English and Chinese guidelines on intravenous ICM use in patients with kidney disease published between January 2018 and June 2023. The quality of the guidelines was assessed with the Scientific, Transparent, and Applicable Rankings (STAR) tool.

**Results:**

Ten guidelines were included, with a median STAR score of 46.0 (range 28.5–61.5). The guidelines performed well in “Recommendations” domain (31/40, 78%), while poor in “Registry” (0/20, 0%) and “Protocol” domains (0/20, 0%). Nine guidelines recommended estimated glomerular filtration rate (eGFR) < 30 mL/min/1.73 m^2^ as the cutoff for referring patients to discuss the risk-benefit balance of ICM administration. Three guidelines further suggested that patients with an eGFR < 45 mL/min/1.73 m^2^ and high-risk factors also need referring. Variable recommendations were seen in the acceptable time interval between renal function test and ICM administration, and that between scan and repeated scan. Nine guidelines recommended to use iso-osmolar or low-osmolar ICM, while no consensus has been reached for the dosing of ICM. Nine guidelines supported hydration after ICM use, but their protocols varied. Drugs or blood purification therapy were not recommended as preventative means.

**Conclusion:**

Guidelines on intravenous ICM use in patients with kidney disease have heterogeneous quality. The scientific societies may consider joint statements on controversial recommendations for variable timing and protocols.

**Critical relevance statement:**

The heterogeneous quality of guidelines, and their controversial recommendations, leave gaps in workflow timing, dosing, and post-administration hydration protocols of contrast-enhanced CT scans for patients with kidney diseases, calling for more evidence to establish a safer and more practicable workflow.

**Key points:**

• Guidelines concerning iodinated contrast media use in kidney disease patients vary.

• Controversy remains in workflow timing, contrast dosing, and post-administration hydration protocols.

• Investigations are encouraged to establish a safer iodinated contrast media use workflow.

**Graphical Abstract:**

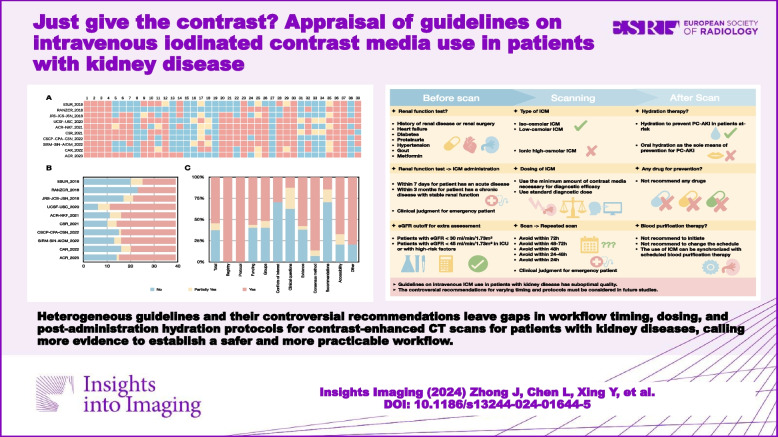

**Supplementary Information:**

The online version contains supplementary material available at 10.1186/s13244-024-01644-5.

## Introduction

Diagnostic imaging with intravenous iodinated contrast media (ICM) is widely used in the clinical practice and provides a large amount of valuable information [1]. The high safety profile guarantees the use of millions of doses of modern ICM worldwide [2–4]. However, intravenous ICM have been historically denied or delayed in patients with kidney diseases due to the concern on the post-contrast acute kidney injury (PC-AKI) [5, 6]. A popular Tiktoker, Dr. Glaucomflecken, has recently uploaded a video on this issue [7]. In the video, the “radiologist” thinks that the contrast will cause further damage to the kidneys, while the “nephrologist” thinks that contrast-induced acute kidney injury is a myth perpetuated by other doctors who do not trust their kidneys — the “nephrologist” even puts up “Just give the contrast” posters everywhere!

Unnecessary delays in diagnostic imaging bring the potential for indirect harm due to delayed diagnosis or misdiagnosis [8–11]. On the other hand, the slogan “Just give the contrast” should not be used for all cases. Clinical practice guidelines serve as an important reference to assist practitioners and patients in appropriate clinical decision-making [12–14]. However, the contradictory comments under the video proved that this problem has not been solved by related guidelines [7, 15]. There is still indistinctness in the use of ICM in patients with kidney diseases in daily practice. It would be necessary to summarize the current guidelines to aid the radiologists and clinicians in balancing the trade-off between the potential risks of intravenous ICM and diagnostic benefits [12, 13].

Therefore, the aim of this study is to perform an appraisal of the guidelines on intravenous ICM use in patients with kidney disease, to highlight the consistencies of the recommendations to inform the best practice and to identify the disagreements among guidelines for consideration in future investigations.

## Materials and methods

### Registration and protocol

The ethical approval or written informed consent was not required for this appraisal of guidelines [16]. The study was conducted and reported according to Preferred Reporting Items for Systematic reviews and Meta-Analyses (PRISMA) statements and checklists (Supplementary Note S1) [17–19]. A protocol has been prospectively drafted and registered to PROSEPRO as CRD42023441532 (Supplementary Note S2) [20]. The guideline search, guideline selection, data extraction, and quality appraisal were duplicated by three independent reviewers (J.Y.Z., L.W.C., and Y.X.). Disagreements were resolved by discussion or consultation with the review group (Y.P.S., Y.B.W., Y.D., R.J., S.L.W., W.J.L., Y.F.H., X.G., D.D.F., H.Z., Y.Z., and W.W.Y.). The statistical analysis was performed by a reviewer (J.Y.Z.) under supervision of a statistical expert (J.J.L.). The synthesis of recommendations was carried out by the whole review group.

### Guideline search and selection

We performed a systematic search to identify guidelines on intravenous iodine contrast media use in patients with kidney disease. We searched six peer-reviewed electronic databases (PubMed, Embase, Web of Science, Cochrane Library, China National Knowledge Infrastructure, Wanfang Data), eight guideline libraries (Guidelines International Network library of guidelines, World Health Organization guidelines, National Institute for Health and Care Excellence, Scottish Intercollegiate Guidelines Network, Canadian Medical Association clinical practice guideline Infobase, New Zealand Guidelines Group, Chinese Medical Ace Base, Practice guideline REgistration for transPAREncy), and ten homepages of radiological societies (International Society of Radiology, European Society of Radiology, Radiological Society of North America, American Roentgen Ray Society, American College of Radiology, Canadian Association of Radiologists, The Royal College of Radiologists, The Royal Australian and New Zealand College of Radiologists, Japan Radiological Society, Chinese Society of Radiology). The selection of the information sources was based on the previous studies and experts’ opinions. The search strategy was developed by a reviewer (J.Y.Z.) using variations of the terms of “contrast media,” “kidney,” and either “guideline,” “consensus,” “statement”. The formal search was conducted until 01 July 2023. The additionally eligible guidelines were distinguished by screening the reference lists of all included guidelines and consulting experts.

We included all the guidelines on ICM in patients with kidney disease. The guidelines were defined as documents that self-identified as a guideline, or a guidance document with recommendations including consensus, appropriateness criteria, manual, etc. [21, 22]. We restricted the publication time from 01 January 2018 onwards to present the recent developments on this topic, and only guidelines written in English and Chinese were available. The following articles were excluded: (1) guidelines developed from the perspective of a medical specialty, in which contrast media were discussed as one of those risk factors for kidney injury [21]; (2) guidelines on intra-arterial contrast media administration, because intra-arterial administration has unique considerations that do not apply to the intravenous route of administration [23]; (3) a previous version of an updated guidelines or a guideline under development; (4) study protocols, primary studies, comments on guidelines, conference abstracts, or other not guidance documents; (5) duplications. The titles and abstracts of unique records were screened, and then their eligibility was confirmed by reading the full texts and supplementary materials. The supplementary materials included but were not limited to protocol, conflict of interest declaration, evidence summary, and dissemination materials. For the guidelines published on multiple journals, all available materials were evaluated as a whole. The search strategy and guideline selection process are presented in Supplementary Note S3.

### Data extraction and quality appraisal

Three independent reviewers extracted the data from all available materials of each guideline according to a predefined data extraction tool (Supplementary Table S1). This tool includes bibliographical information, characteristics, and key recommendations. The same three reviewers independently evaluated the quality of included guidelines by using the Scientific, Transparent and Applicable Rankings (STAR) tool (Supplementary Table S2) [24]. This tool not only covers the domains in the existing Appraisal of Guidelines for Research and Evaluation II (AGREE-II) [25] and Reporting Items for Practice Guidelines in Healthcare (RIGHT) [26] tool, but also includes extra elements of applicability, development transparency, and prospective registries, to allow a comprehensive evaluation [27–30]. The STAR tool has been validated by evaluating hundreds of guidelines and consensuses [28, 29] and is suitable for our study. The STAR tool includes 39 items in 11 domains. The items were rated as 1 for full adherence, 0.5 for partial adherence, and 0 for not adherent at all. The sum STAR score was calculated as the sum of domain weight × item weight × item score of 37 items, with a maximum sum score of 100. A guideline with a higher score is considered to be better in quality. Before the formal data extraction and quality appraisal, the reviewers tested and modified the tools to reach a shared operation of each item [31]. The discussed items and reached consensus are available in Supplementary Note S4.

### Data analysis

The statistical analysis was performed with R language version 4.1.3 within RStudio version 3.6.3 by using relevant packages [32]. The key recommendations from the included guidelines were qualitatively summarized by consensus conferences. The data analysis process is available in Supplementary Note S5.

## Results

### Guideline search and selection

**Fig. 1:**
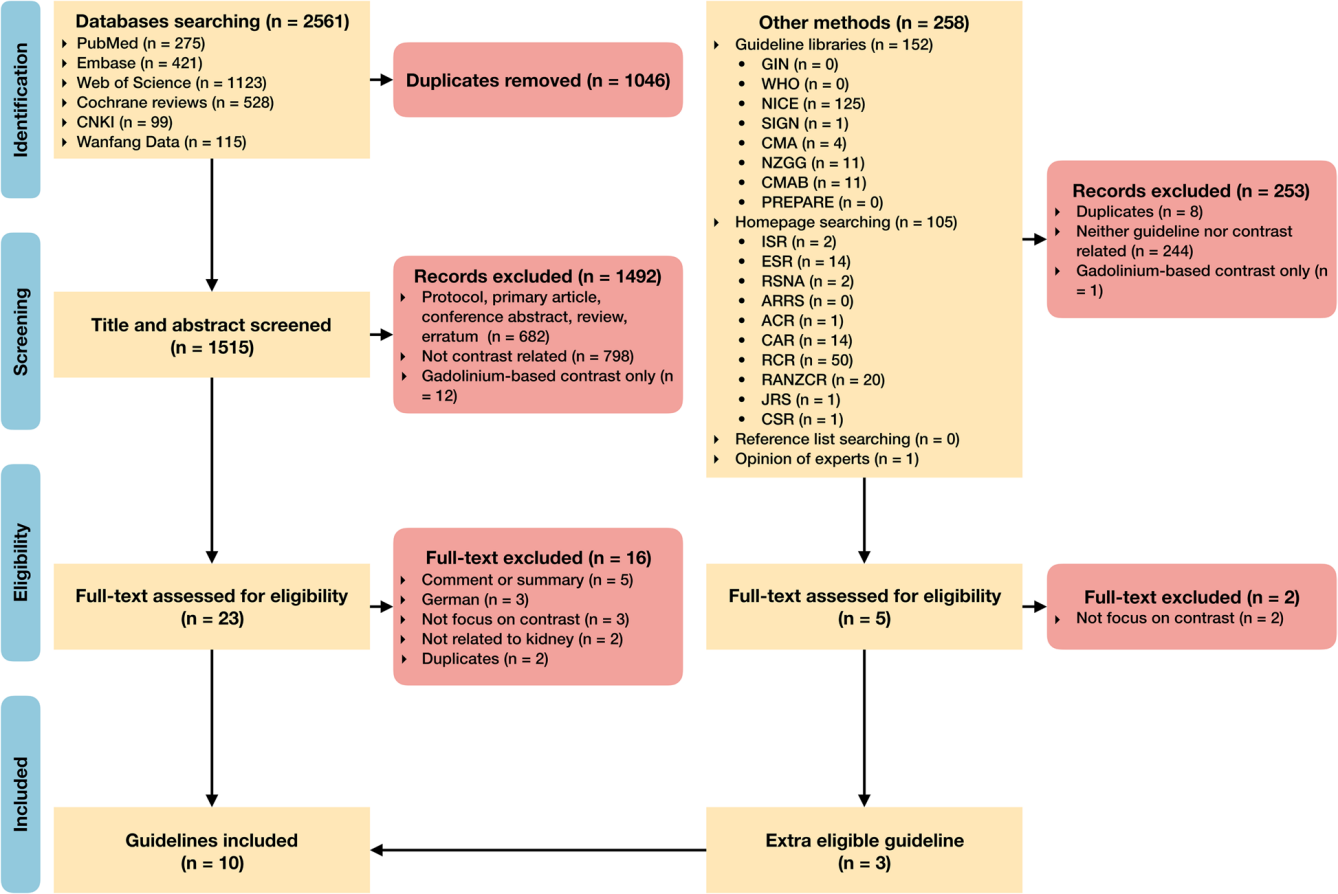
Flowchart of guideline search and selection. CNKI = China National Knowledge Infrastructure, GIN = Guidelines International Network library of guidelines, WHO = World Health Organization guidelines, NICE = National Institute for Health and Care Excellence, SIGN = Scottish Intercollegiate Guidelines Network, CMA = Canadian Medical Association clinical practice guideline Infobase, NZGG = New Zealand Guidelines Group, CMAB = Chinese Medical Ace Base, PREPARE = Practice guideline Registration for transparency, ISR = International Society of Radiology, ESR = European Society of Radiology, RSNA = Radiological Society of North America, ARRS = American Roentgen Ray Society, ACR = American College of Radiology, CAR = Canadian Association of Radiologists, RCR = The Royal College of Radiologists, RANZCR = The Royal Australian and New Zealand College of Radiologists, JRS = Japan Radiological Society, CSR = Chinese Society of Radiology

The systematic search identified 2561 records from all the information sources in total. After screening the titles and abstracts of 1515 unique records, 23 full texts, and their supplementary materials were retrieved for eligibility assessment, in which 7 were considered as eligible. After searching of guideline libraries and homepages of radiological societies, 3 extra eligible guidelines were identified. Reference list screening and consultation with experts did not find additional eligible guidelines. Eventually, 10 guidelines were included [33–42] (Fig. 1). The excluded records of full texts are listed in Supplementary Note S6.

### Guideline characteristics

There were 5/10 guidelines developed by radiological societies alone [33, 34, 38, 41, 42], 3/10 by both radiological societies and clinical medical societies [35, 37, 40], 1/10 by clinical medical societies alone [39], and 1/10 by universities [36]. There were 6/10 guidelines developed specially for contrast media use in patients with kidney diseases [35, 37–41], while 4/10 were guidelines for intravenous contrast use [33, 34, 36, 42]. The characteristics of the guidelines are listed in Table 1.

**Table 1:** General characteristics of guidelines

**Guideline**	**Reference**	**Organization(s)**	**Year**	**Region**	**STAR score**
ESUR_2018	[33]	European Society of Urogenital Radiology	2018	Europe	61.5
RANZCR_2018	[34]	Royal Australian and New Zealand College of Radiologists	2018	Australian and New Zealand	58.7
JRS-JCS-JSN_2018	[35]	Japanese Society of Nephrology, Japan Radiological Society, Japanese Circulation Society	2018	Japan	57.5
UCSF-USC_2020	[36]	University of California San Francisco, University of Southern California	2020	USA	28.5
ACR-NKF_2021	[37]	American College of Radiology, National Kidney Foundation	2021	USA	38.0
CSR_2021	[38]	Chinese Society of Radiology	2021	China	37.3
CSCP-CPA-CSN_2022	[39]	Chinese Society of Clinical Pharmacy, Chinese Pharmaceutical Association, Chinese Society of Nephrology	2022	China	47.5
SIRM-SIN-AIOM_2022	[40]	Italian College of Radiology, Italian College of Nephrology, Italian Association of Medical Oncology	2022	Italia	39.4
CAR_2022	[41]	Canadian Association of Radiologists	2022	Canada	46.3
ACR_2023	[42]	American College of Radiology	2023	USA	45.6

*ACR* American College of Radiology, *ACR-NKF* American College of Radiology, and National Kidney Foundation,*CAR* Canadian Association of Radiologists, *CSCP-CPA-CSN* Chinese Society of Clinical Pharmacy, Chinese Pharmaceutical Association, and Chinese Society of Nephrology, *CSR* Chinese Society of Radiology, *ESUR* European Society of Urogenital Radiology, *JRS-JCS-JSN* Japan Radiological Society, Japanese Circulation Society, and Japanese Society of Nephrology, *RANZCR* Royal Australian and New Zealand College of Radiologists, *SIRM- SIN-AIOM* Italian College of Radiology, Italian College of Nephrology, and Italian Association of Medical Oncology, *UCSF-USC* University of California San Francisco, and University of Southern California

### Guideline quality assessment

**Fig. 2:**
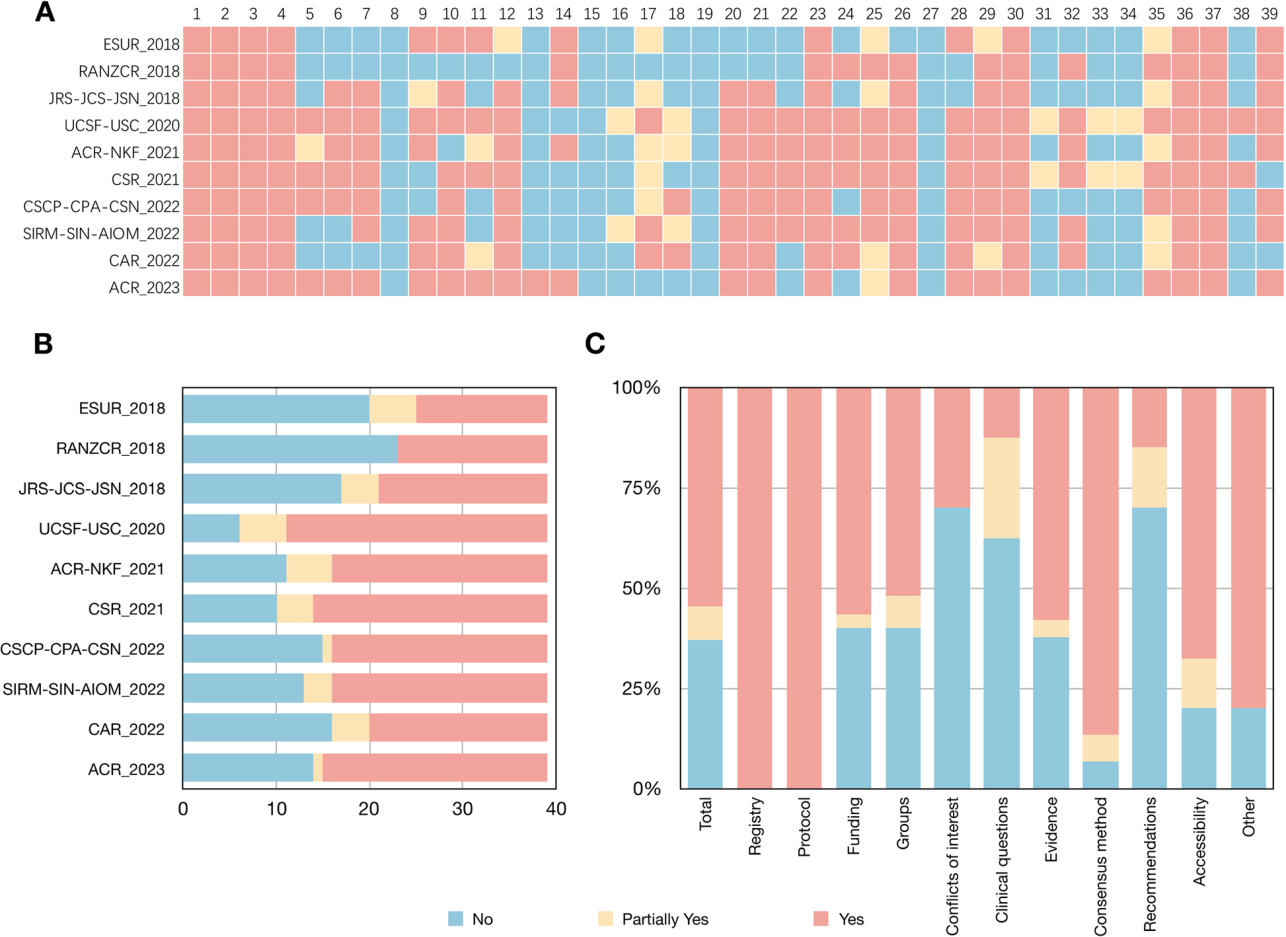
Quality appraisal of guidelines using the STAR tool. **A** STAR item rating of each guideline. **B** STAR rating of each domain. **C** STAR rating of each guideline. STAR = Scientific, Transparent and Applicable Rankings tool. ACR = American College of Radiology; ACR-NKF = American College of Radiology, and National Kidney Foundation; CAR = Canadian Association of Radiologists; CSCP-CPA-CSN = Chinese Society of Clinical Pharmacy, Chinese Pharmaceutical Association, and Chinese Society of Nephrology; CSR = Chinese Society of Radiology; ESUR = European Society of Urogenital Radiology; JRS-JCS-JSN = Japan Radiological Society, Japanese Circulation Society, and Japanese Society of Nephrology; RANZCR = Royal Australian and New Zealand College of Radiologists; SIRM- SIN-AIOM = Italian College of Radiology, Italian College of Nephrology, and Italian Association of Medical Oncology; UCSF-USC = University of California San Francisco, and University of Southern California

The median (range) of the sum STAR score for the included guidelines was 46.0 (28.5–61.5) (Table 1). There were 145, 32, and 213 items that were rated as “Yes,” “Partially yes,” and “No” for STAR, respectively (Fig. 2). The guideline developed by the Royal Australian and New Zealand College of Radiologists showed the highest quality [34]. In contrast, the guideline developed by universities were with lowest quality [36]. The domains of “Recommendations” (Domain 9, 31/40, 78%), “Clinical questions” (Domain 6, 30/40, 75%), and “Conflicts of interest” (Domain 5, 14/20, 70%) had the highest ratings, while the lowest scores were in the domains of “Consensus method” (Domain 8, 3/30, 10%), “Registry” (Domain 1, 0/20, 0%), and “Protocol” (Domain 2, 0/20, 0%) (Table 2 and Supplementary Table S3). The remaining domains gained only less than a half of scores.

**Table 2:** Quality appraisal of guidelines using the STAR tool

**Item**	**Item score**	**Rating* n/N*** **(%)**
**Overall**	**100.0**	**161/390 (41)**
**Domain 1: Registry**	**5.0**	**0/20 (0)**
1. Register the guideline on an appropriate platform.	1.5	0/10 (0)
2. Provide information about the registry platform and registry ID of the guideline.	3.5	0/10 (0)
**Domain 2: Protocol**	**5.0**	**0/20 (0)**
3. Provide details of the guideline protocol.	1.9	0/10 (0)
4. Identify how the guideline protocol is accessible from an open-source platform (e.g., guideline registry platform or website).	3.1	0/10 (0)
**Domain 3: Funding**	**3.2**	**12.5/30 (42)**
5. Describe the sources of funding for the development of the guideline.	1.0	5.5/10 (55)
6. Describe the role of funder(s) in the guideline development.	0.9	4/10 (40)
7. Declare that the funder(s) did not influence the guideline’s recommendations.	1.3	3/10 (30)
**Domain 4: Guideline development groups**	**7.3**	**22/50 (44)**
8. List the institutional affiliations of all individuals involved in developing the guideline.	0.9	10/10 (100)
9. Describe the composition of the development groups.	1.0	3.5/10 (35)
10. Describe the responsibilities of all individuals or sub-groups involved in developing the guideline.	1.3	2/10 (20)
11. Identify experts from at least two disciplines in addition to the guideline’s topic who took part in the development.	1.3	5/10 (50)
12. Identify guideline methodologists or experts in evidence-based medicine who took part in the development.	2.8	1.5/10 (15)
**Domain 5: Conflicts of interest**	**9.2**	**14/20 (70)**
13. Describe whether conflicts of interest existed.	4.4	9/10 (90)
14. Indicate information about the evaluation and management of conflicts of interest.	4.8	5/10 (50)
**Domain 6: Clinical questions**	**8.9**	**30/40 (75)**
15. Identify the clinical questions that the guideline focuses on.	6.4	10/10 (100)
16. Introduce the methods of collecting clinical questions, such as literature search, survey of users, or consultation of experts.	2.5	9/10 (90)
17. Indicate how the clinical questions were selected and sorted.	3.4	6.5/10 (65)
18. Format clinical questions in PICO (population/patients, intervention, control/comparator, and outcome) or other formats.	4.8	4.5/10 (45)
**Domain 7: Evidence**	**25.1**	**36/90 (40)**
19. Identify the references for evidence supporting the main recommendations.	1.7	10/10 (100)
20. State to the details of the systematic search (e.g., names of databases, selection criteria, search strategies).	2.2	2/10 (20)
21. Indicate the inclusion and exclusion criteria of research evidence.	1.5	2/10 (20)
22. Assess the risk of bias or methodological quality of the included studies.	1.9	5/10 (50)
23. Summarize and analyze the research evidence.	2.1	0/10 (0)
24. Indicate the standard used to grade the evidence quality.	2.2	4/10 (40)
25. Provide the GRADE evidence profile or summary of the results of evidence grading.	2.4	2/10 (20)
26. Provide reference to the full text of systematic reviews.	1.7	1/10 (10)
27. Identify the clinical questions with insufficient evidence (low quality) and indicate future research directions to collect more evidence.	1.2	10/10 (100)
**Domain 8: Consensus method**	**10.7**	**3/30 (10)**
28. Indicate the specific method(s) used to reach consensus (e. g., the Delphi method, Nominal group technique, or informal approaches).	5.1	2/10 (20)
29. Describe the criteria to inform decisions other than the certainty of the evidence (e.g., resource requirements, preferences and values of patients, cost–benefit balance, accessibility, health equity, acceptability). 30. Provide the records of the consensus process.	3.8	1/10 (10)
30. Provide the records of the consensus process.	1.8	0/10 (0)
**Domain 9: Recommendations**	**17.1**	**31/40 (78)**
31. Make the recommendations clearly identifiable (e.g., in a table, or using enlarged or bold fonts).	4.1	9/10 (90)
32. Indicate the strength of all recommendations.	6.3	4/10 (40)
33. Provide the explanations for all recommendations.	3.9	9/10 (90)
34. Indicate the considerations (e.g., adverse effects) in clinical practice when implementing the recommendations.	2.8	9/10 (90)
**Domain 10: Accessibility**	**7.3**	**10.5/40 (26)**
35. Make the guideline accessible through multiple platforms (e. g., guideline libraries, conference presentations, and websites).	2.5	2.5/10 (25)
36. Provide tailored editions of the guidelines for different groups of target users (e.g., patients, public, primary care physicians).	1.4	0/10 (0)
37. Present the guideline or recommendations visually, such as with figures or videos.	1.1	0/10 (0)
38. Make the full guideline downloadable free of charge.	2.3	8/10 (80)
**Domain 11: Other**	**1.2**	**2/10 (20)**
39. Provide a flowchart of clinical pathways reflecting the recommendations.	1.2	2/10 (20)

### Synthesis of recommendations

**Fig. 3:**
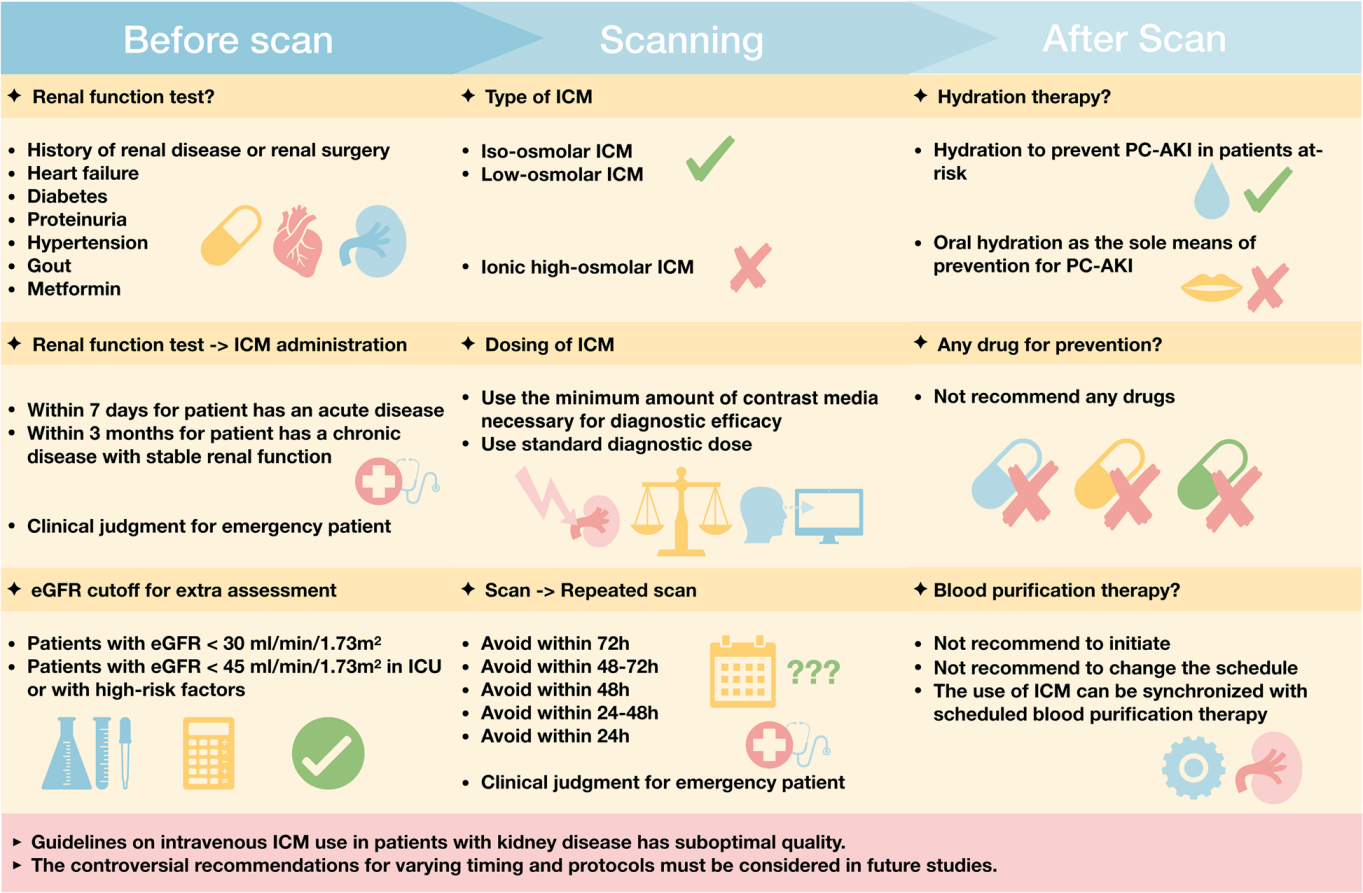
Summary of key recommendations. The key recommendations are summarized according to three stages of contrast-enhanced CT scan. Note not all the recommendations are listed. eGFR = estimated glomerular filtration rate, ICM = iodinated contrast media, PC-AKI = post-contrast acute kidney injury

The recommendations for the ICM use in patients with kidney disease were compared (Fig. 3 and Table 3). The discussed recommendations are listed in Supplementary Tables S4 and S5.

**Table 3:** Summary of key recommendations

**Questions and answers**	**Guidelines**
**Who should undergo renal function test before contrast media administration?**	
History of renal disease or renal surgery	ESUR_2018 [33], RANZCR_2018 [34], UCSF-USC_2020 [36], ACR, NKF_2021 [37], CSR_2021 [38], CAR_2022 [41], ACR_2023 [42]
Heart failure	ESUR_2018 [33]
Diabetes	ESUR_2018 [33], RANZCR_2018 [34], UCSF-USC_2020 [36]
Proteinuria	ESUR_2018 [33]
Hypertension	ESUR_2018 [33], UCSF-USC_2020 [36]
Gout	ESUR_2018 [33]
Metformin	RANZCR_2018 [34], ACR-NKF_2021 [37], ACR_2023 [42]
Aging	UCSF-USC_2020 [36], CSCP-CPA-CSN_2022 [39]
Against Aging	RANZCR_2018 [34]
**How long is the time interval between renal function test and administration acceptable?**	
Within 7 days for patient has an acute disease; within 3 months for patient has a chronic disease with stable renal function	ESUR_2018 [33], JRS-JCS-JSN_2018 [35], CSR_2021 [38], SIRM-SIN-AIOM_2022 [40]
Within 6 weeks for outpatients, within 7 days for inpatients with renal impairment.	UCSF-USC_2020 [36]
Within 7 days for non-emergency patients.	CSCP-CPA-CSN_2022 [39]
Within 7 days for inpatients or emergency patients.	CAR_2022 [41]
Depends on clinical judgment.	RANZCR_2018 [34], ACR_2023 [42]
**What is the eGFR cutoff for patients at risk for PC-AKI/ needs referring/ further treatment?**	
Patients with eGFR < 30 mL/min/1.73 m^2^	ESUR_2018 [33], RANZCR_2018 [34], JRS-JCS-JSN_2018 [35], UCSF-USC_2020 [36], ACR-NKF_2021 [37], CSCP-CPA-CSN_2022 [39], SIRM-SIN-AIOM_2022 [40], CAR_2022 [41], ACR_2023 [42]
Patients with eGFR < 45 mL/min/1.73 m^2^ in ICU or with high-risk factors	ESUR_2018 [33], CSR_2021 [38], SIRM-SIN-AIOM_2022 [40]
**What kind of contrast media is recommended for high-risk patients?**	
Support the use of iso-osmolar ICM and low-osmolar ICM.	ESUR_2018 [33], JRS-JCS-JSN_2018 [35], ACR-NKF_2021 [37], CSR_2021 [38], CSCP-CPA-CSN_2022 [39], CAR_2022 [41], ACR_2023 [42]
Against the use of ionic high-osmolar ICM.	ESUR_2018 [33], ACR-NKF_2021 [37], CSR_2021 [38], CSCP-CPA-CSN_2022 [39]
**Is reduced contrast media dosage recommended for high-risk patients?**	
Use the minimum amount of contrast media necessary for diagnostic efficacy.	ESUR_2018 [33], JRS-JCS-JSN_2018 [35], CSR_2021 [38], CSCP-CPA-CSN_2022 [39], SIRM-SIN-AIOM_2022 [40]
Use standard diagnostic dose.	ACR-NKF_2021 [37], CAR_2022 [41], ACR_2023 [42]
**How long is the suitable time interval between scan and repeated scan?**	
Repeated CM injections should be avoided within 72 h.	CSR_2021 [38]
Repeated CM injections should be avoided within 48-72 h.	ESUR_2018 [33], CSCP-CPA-CSN_2022 [39]
Repeated CM injections should be avoided within 48 h.	CAR_2022 [41]
Repeated CM injections should be avoided within 24-48 h.	JRS-JCS-JSN_2018 [35]
Repeated CM injections should be avoided within 24 h.	ACR_2023 [42]
**Is the hydration recommend for high-risk patients?**	
Support hydration to prevent PC-AKI in patients at-risk.	ESUR_2018 [33], RANZCR_2018 [34], JRS-JCS-JSN_2018 [35], UCSF-USC_2020 [36], ACR-NKF_2021 [37], CSR_2021 [38], CSCP-CPA-CSN_2022 [39], SIRM-SIN-AIOM_2022 [40], ACR_2023 [42]
Against oral hydration as the sole means of prevention for PC-AKI.	ESUR_2018 [33], JRS-JCS-JSN_2018 [35], CSCP-CPA-CSN_2022 [39]
**Is any drug recommend for high-risk patients, and what are they?**	
Not recommend any drugs to prevent PC-AKI in patients at-risk.	ESUR_2018 [33], RANZCR_2018 [34], JRS-JCS-JSN_2018 [35], ACR-NKF_2021 [37], CSR_2021 [38], CSCP-CPA-CSN_2022 [39], SIRM-SIN-AIOM_2022 [40], CAR_2022 [41], ACR_2023 [42]
**Is the blood purification therapy recommend for high-risk patients?**	
Not recommend to initiate blood purification therapy.	ESUR_2018 [33], JRS-JCS-JSN_2018 [35], UCSF-USC_2020 [36], ACR-NKF_2021 [37], CSR_2021 [38], CSCP-CPA-CSN_2022 [39], CAR_2022 [41], ACR_2023 [42]
Not recommend to change the schedule of blood purification therapy.	ACR-NKF_2021 [37], SIRM-SIN-AIOM_2022 [40]
The use of ICM can be synchronized with scheduled blood purification therapy.	CSR_2021 [38]

*ACR* American College of Radiology, *ACR-NKF* American College of Radiology, and National Kidney Foundation, *CAR* Canadian Association of Radiologists, *CSCP-CPA-CSN* Chinese Society of Clinical Pharmacy, Chinese Pharmaceutical Association, and Chinese Society of Nephrology, *CSR* Chinese Society of Radiology, *ESUR* European Society of Urogenital Radiology, *JRS-JCS-JSN* Japan Radiological Society, Japanese Circulation Society, and Japanese Society of Nephrology, *RANZCR* Royal Australian and New Zealand College of Radiologists, *SIRM- SIN-AIOM* Italian College of Radiology, Italian College of Nephrology, and Italian Association of Medical Oncology, *UCSF-USC* University of California San Francisco, and University of Southern California

To identify whether the patient needs renal function testing, most of the guidelines emphasized the history of kidney disease [33, 34, 36–38, 41, 42]. Other potential risk factors that should be considered were diabetes [33, 34, 36], metformin use [34, 37, 42], hypertension [33, 36], heart failure [33], proteinuria [33], and gout [33]. Aging was considered for renal function testing by two guidelines [36, 39]. However, another guideline was against it, treating renal function reduction as normal physiological changes with aging [34]. Questionnaires, risk models, and stratification tools showed good performance in predicting the risk of PC-AKI, but there was no consensus achieved on which one to be used in clinical practice [33, 36, 39–41]. The acceptable interval between renal function testing and ICM administration varied among guidelines. Three guidelines [35, 38, 40] followed the earlier guideline [33] to recommend renal function test within 7 days for a patient who has an acute disease, and within 3 months for a patient who has a chronic disease with stable renal function, while other guidelines recommended 7 days to 6 weeks as appropriate time intervals depending on the clinical judgment [34, 36, 39, 41, 42]. Nine guidelines agreed that patients with eGFR < 30 mL/min/1.73 m^2^ are at risk for PC-AKI, or need referring and further treatment [33–37, 39–42]. In addition to the patients with eGFR < 30 mL/min/1.73 m^2^, three guidelines considered patients with eGFR < 45 mL/min/1.73 m^2^ in intensive care unit or with high-risk factors to also be at-risk for PC-AKI [33, 38, 40]. The calculation of eGFR based on serum creatinine was considered as the commonly available method [33–35, 37, 38, 40, 42] (Supplementary Table S6).

The iso-osmolar ICM and low-osmolar ICM were recommended for contrast-enhanced CT scans for patients with or without kidney diseases [33, 35, 37–39, 41, 42]. None of the guidelines recommended a specific type of ICM. One guideline suggested that decisions about the use of low-osmolar or iso-osmolar ICM should be made based on factors such as cost and availability [41]. In contrast, the use of ionic high-osmolar ICM was not recommended due to the relatively high risk for adverse effects [33, 37–39]. Five guidelines recommended to use the minimum amount of contrast media necessary for diagnostic efficacy [33, 35, 38–40], while three guidelines recommended to use of standard diagnostic doses [37, 41, 42]. The suitable time interval recommended for the initial contrast-enhanced CT scan and the repeated scan differed widely among guidelines from 24 h to 72 h [33, 35, 38, 39, 41, 42]. However, the repeated contrast-enhanced CT scans were not forbidden if it is clinically necessary.

Nine guidelines supported hydration as a preventative mean for PC-AKI [33–40, 42]. Intravenous hydration was considered as the standard selection, but the protocols varied among guidelines and needed individualization according to the patients. Three guidelines obviously argued against oral hydration as the sole means of prevention for PC-AKI [33, 35, 39]. It is of note that one guideline made no recommendation on hydration, noting a lack of evidence on benefits [41]. This guideline suggested that institutions choose practices best suited to the local environments regarding the use of hydration or not, and the protocols for hydration were left to the judgment of the practitioner. If hydration is considered to be necessary for the patient, the discrepancy in recommended protocols should be noted [33–40, 42]. Intravenous fluid is usually saline 0.9% or sodium bicarbonate 1.4%. The recommendations on timing of hydration ranged from 1 to 12 h before the ICM use, and from 1 to 12 h after the ICM use. The volume of hydration was recommended to be a fixed volume of 500 mL before and after the use of ICM or adjusted according to the body weight. However, the practitioners should individualize preventative hydration in patients with risk of hydration, such as severe congestive heart failure. The guidelines recommended neither drugs to prevent PC-AKI [33–35, 37–42], nor initiation of the blood purification therapy [33, 35–39, 41, 42]. Two guidelines did not recommend to change the schedule of the blood purification therapy to adapt the contrast-enhanced CT scan [37, 40]. On the other hand, one guideline declared that the contrast-enhanced CT scan can be synchronized with the already scheduled blood purification therapy [38].

## Discussion

This study systematically appraised the guidelines on intravenous ICM use concerning kidney disease. The overall quality of the included guidelines is heterogeneous. Our study showed that the guidelines have almost reached consensus in the eGFR cutoff for referring patients to discuss the risk-benefit balance of ICM administration before scanning, the type of ICM to use for the scan, and the hydration therapy for reducing PC-AKI after a scan. However, the recommendations were still variable among guidelines for the acceptable time interval between renal function test and ICM administration, the shortest time interval between scan and re-scan, dosing of ICM, and protocols for hydration therapy.

We used the STAR tool to comprehensively assess the quality of the included guidelines. The “Registry” and “Protocol” of guidelines have potential in reducing duplication, improving collaboration, and increasing transparency [43]. These two domains were firstly added in the STAR tool, but not mentioned in the AGREE-II and RIGHT tools [24–26]. Since all the guidelines were developed before the STAR tool, it is not strange that all the included guidelines did not provide the register and protocol information. We encourage future guidelines to be registered before development and provide a protocol to guarantee the rigor of development. The “Recommendations” and “Clinical questions” were with high adherence rates. This allows the radiologists and clinicians to accurately identify the relevant recommendations. However, the “Other” domain indicated that only two guidelines provided a flowchart of clinical pathways reflecting the recommendations [38, 41], which potentially hindered the clinicians to reach a visual understanding of the guidelines. The low rating in “Accessibility” also calls for more efforts on the dissemination to allow more stakeholders to be aware of the guidelines and change the practice in daily radiological workflows. The rigorous methodologies and strategies should be used to provide a solid foundation for overall credibility and quality during the guideline development in order to improve the reliability and rationality of recommendations [44]. The methodological quality of the guideline was related to the “Guideline development groups”, “Evidence”, and “Consensus method” domains in the STAR tool. However, these domains were with low scoring, indicating a lack of attention and reporting of the methodological aspect.

There were several controversial recommendations that must be considered in future studies. First, the Choyke questionnaire may work well for the selection of patients to undergo serum creatinine [45]. However, many hospitals measure serum creatinine in all patients scheduled for intravenous ICM use since eGFR can detect more patients with kidney diseases than questionnaires [46]. On the other hand, many risk models and tools have been developed for PC-AKI prediction [33, 36, 39–41]. The validation studies were still lacking for the selection of these risk models to guide renal function testing [47–49]. Second, it is still dependent on clinical judgment whether repeated renal function is necessary or emergency contrast‐enhanced CT without renal function results are appropriate [34, 50, 51]. In non-emergency situations, the acceptable time interval between renal function test and ICM administration are either not mentioned or varied between guidelines. There is still a need to establish a shared consensus on this issue to guide the clinical practice. Currently, the recommendations from the European Society of Urogenital Radiology were most widely accepted [34, 35, 38, 40, 52]. Third, the shortest available time interval between repeated scans is also undetermined. The American College of Radiology recommended that the time interval of repeated ICM injections was at least 24 h, which is the shortest among guidelines in which this was included [42]. The most conservative suggestion was provided by the Chinese Society of Radiology [38] that recommended avoiding repeated ICM injections within 72 h. Likewise, guidelines still value the clinical judgment in the face of life-threatening illness, allowing repeated scans to establish a confident diagnosis and treatment plan [41]. Fourth, the current study only concerned the use of intravenous ICM. It remains unknown whether the schedule of contrast-enhanced CT is reasonable after intra-arterial ICM use or gadolinium-based contrast media use. All the guidelines did not recommend reducing the dose of ICM for high-risk patients at expense of image quality, but it might be reasonable to use the minimum amount of contrast media to satisfy the diagnostic efficacy. Further studies are encouraged to apply advanced acquisition and reconstruction techniques to reduce the requisite ICM dose [53–56, 62]. A promising measure of systemic ICM exposure is the contrast-dose/absolute GFR ratio [57]. The measure may serve as a useful tool in determining whether the use of ICM is safe and appropriate. The potential of this measure in predicting the risk of PC-AKI after contrast-enhanced CT and intra-arterial examinations should be evaluated by prospective studies. Fifth, the protocols of hydration therapy for patients at risk vary among guidelines. It is difficult here to make recommendations for protocol selection. Nevertheless, the guidelines agreed that the specific hydration therapy for each patient should be personalized by clinical justification. The details of the hydration therapy protocol need further investigation including whether intravenous and oral hydration therapy should be conducted [58], which solution should be used for intravenous hydration therapy, as well as the timing, volume, and speed of hydration therapy.

The following limitations of this study should be addressed. First, our study did not include guidelines written in languages other than English or Chinese. Although we searched multiple databases and guideline libraries, our study did not present various viewpoints from all stakeholders, and therefore may still have bias. Second, the STAR tool was a recently developed tool without wide validation compared to the AGREE-II and RIGHT tool. This tool has good reliability, validity, and efficiency [24], and has been validated in evaluations of hundreds of Chinese guidelines [28, 29]. The weights of domains and items of STAR were subjectively determined, and the total score may be sensitive to the weighting [24]. Nevertheless, this tool is still a timely tool for comprehensive evaluation of guidelines. Third, the summary of the recommendations was not reached by using an anonymous Delphi process. Our consensus conference may introduce bias due to the dominance of some participants and confirmation pressure [59, 60]. However, direct interactions among participants are more likely to allow participants to reach a consensus and deepen their understanding of the reasons for disagreement [61]. Finally, our study only identified discrepancies between guidelines, but did not address them. Further investigations were encouraged to generate robust evidence for solving the discrepancies. Therefore, the current recommendations must be interpreted with caution.

To summarize, the quality of the included guidelines was heterogeneous. The “Just give the contrast” slogan should be interpreted with caution. Most guidelines showed consistent recommended eGFR < 30 mL/min/1.73 m^2^ as the cutoff for referring patients to discuss the risk-benefit balance of ICM administration before a scan, use of iso-osmolar or low-osmolar ICM for scan, and hydration therapy after a scan. However, there are variable recommendations on the acceptable time interval between renal function test and ICM administration, the shortest time interval between scan and re-scan, dosing of ICM, and protocols for hydration therapy. These gaps need to be considered in future studies.

### Supplementary Information

**Additional file 1:** **Supplementary Note S1.** PRISMA checklists. **Supplementary Note S2.** Review protocol.**Supplementary Note S3.** Search strategy and study selection. **Supplementary Note S4.** Data extraction and quality appraisal. **Supplementary Note S5.** Data analysis process. **Supplementary Note S6.** Excluded records of full-texts with justifications. **Supplementary Table S1.** Data extraction tool. **Supplementary Table S2.** STAR tool checklist. **Supplementary Table S3.** STAR rating of each guideline. **Supplementary Table S4.** List of discussed recommendations. **Supplementary Table S5.** List of answers for interested questions. **Supplementary Table S6.** Formula for eGFR calculation.

## Data Availability

All data generated or analyzed during this study are included in this published article and its supplementary information files.

## Abbreviations

AGREE-II: Appraisal of Guidelines for Research and Evaluation II
eGFR: Estimated glomerular filtration rate
ICM: Iodinated contrast media
PC-AKI: Post-contrast acute kidney injury
PRISMA: Preferred Reporting Items for Systematic reviews and Meta-Analyses
RIGHT: Reporting Items for Practice Guidelines in Healthcare
STAR: Scientific, Transparent and Applicable Rankings tool
