# Supplementary material for: Just give the contrast? Appraisal of guidelines on intravenous iodinated contrast media use in patients with kidney disease
Source: Insights Imaging. 2024 Mar 18;15:77. doi: 10.1186/s13244-024-01644-5 (PMC10948651; doi:10.1186/s13244-024-01644-5)
Supplement: Supplementary file 1 — Additional file 1: Supplementary Note S1. PRISMA checklists. Supplementary Note S2. Review protocol.Supplementary Note S3. Search strategy and study selection. Supplementary Note S4. Data extraction and quality appraisal. Supplementary Note S5. Data analysis process. Supplementary Note S6. Excluded records of full-texts with justifications. Supplementary Table S1. Data extraction tool. Supplementary Table S2. STAR tool checklist. Supplementary Table S3. STAR rating of each guideline. Supplementary Table S4. List of discussed recommendations. Supplementary Table S5. List of answers for interested questions. Supplementary Table S6. Formula for eGFR calculation. [file 13244_2024_1644_MOESM1_ESM.pdf]

Just give the contrast? Appraisal of guidelines on intravenous iodinated contrast media use in patients with kidney disease

ELECTRONIC SUPPLEMENTARY MATERIAL

List of Supplementary Materials

Supplementary Note S1 PRISMA checklists
Supplementary Note S2 Review protocol
Supplementary Note S3 Search strategy and study selection
Supplementary Note S4 Data extraction and quality appraisal
Supplementary Note S5 Data analysis process
Supplementary Note S6 Excluded records of full-texts with justifications

Supplementary Table S1 Data extraction tool
Supplementary Table S2 STAR tool checklist
Supplementary Table S3 STAR rating of each guideline
Supplementary Table S4 List of discussed recommendations
Supplementary Table S5 List of answers for interested questions
Supplementary Table S6 Formula for eGFR calculation

Supplementary Note S1 PRISMA checklists

1. PRISMA-P checklist

**Section and topic**	**No**	**Checklist item**	**Reported**
**ADMINISTRATIVE INFORMATION**			
Title:			
Identification	1a	Identify the report as a protocol of a systematic review	Supplementary Note S2
Update	1b	If the protocol is for an update of a previous systematic review, identify as such	Supplementary Note S2
Registration	2	If registered, provide the name of the registry (such as PROSPERO) and registration number	Supplementary Note S2
Authors:			
Contact	3a	Provide name, institutional affiliation, e-mail address of all protocol authors; provide physical mailing address of corresponding author	Supplementary Note S2
Contributions	3b	Describe contributions of protocol authors and identify the guarantor of the review	Supplementary Note S2
Amendments	4	If the protocol represents an amendment of a previously completed or published protocol, identify as such and list changes; otherwise, state plan for documenting important protocol amendments	Supplementary Note S2
Support:			
Sources	5a	Indicate sources of financial or other support for the review	Supplementary Note S2
Sponsor	5b	Provide name for the review funder and/or sponsor	Supplementary Note S2
Role of sponsor or funder	5c	Describe roles of funder(s), sponsor(s), and/or institution(s), if any, in developing the protocol	Supplementary Note S2
**INTRODUCTION**			
Rationale	6	Describe the rationale for the review in the context of what is already known	Supplementary Note S2
Objectives	7	Provide an explicit statement of the question(s) the review will address with reference to participants, interventions, comparators, and outcomes (PICO)	Supplementary Note S2
**METHODS**			
Eligibility criteria	8	Specify the study characteristics (such as PICO, study design, setting, time frame) and report characteristics (such as years considered, language, publication status) to be used as criteria for eligibility for the review	Supplementary Note S2
Information sources	9	Describe all intended information sources (such as electronic databases, contact with study authors, trial registers or other grey literature sources) with planned dates of coverage	Supplementary Note S2
Search strategy	10	Present draft of search strategy to be used for at least one electronic database, including planned limits, such that it could be repeated	Supplementary Note S2 and S3
Study records:			
Data management	11a	Describe the mechanism(s) that will be used to manage records and data throughout the review	Supplementary Note S2
Selection process	11b	State the process that will be used for selecting studies (such as two independent reviewers) through each phase of the review (that is, screening, eligibility and inclusion in meta-analysis)	Supplementary Note S2 and S3

Data collection process	11c	Describe planned method of extracting data from reports (such as piloting forms, done independently, in duplicate), any processes for obtaining and confirming data from investigators	Supplementary Note S2 and S4
Data items	12	List and define all variables for which data will be sought (such as PICO items, funding sources), any pre-planned data assumptions and simplifications	Supplementary Note S2 and S4
Outcomes and prioritization	13	List and define all outcomes for which data will be sought, including prioritization of main and additional outcomes, with rationale	Supplementary Note S2 and S4
Risk of bias in individual studies	14	Describe anticipated methods for assessing risk of bias of individual studies, including whether this will be done at the outcome or study level, or both; state how this information will be used in data synthesis	Supplementary Note S2 and S4
Data synthesis	15a	Describe criteria under which study data will be quantitatively synthesised	Supplementary Note S2 and S5
	15b	If data are appropriate for quantitative synthesis, describe planned summary measures, methods of handling data and methods of combining data from studies, including any planned exploration of consistency (such as I^2, Kendall's τ)	Supplementary Note S2 and S5
	15c	Describe any proposed additional analyses (such as sensitivity or subgroup analyses, meta-regression)	Not applicable
	15d	If quantitative synthesis is not appropriate, describe the type of summary planned	Supplementary Note S2 and S5
Meta-bias(es)	16	Specify any planned assessment of meta-bias(es) (such as publication bias across studies, selective reporting within studies)	Not applicable
Confidence in cumulative evidence	17	Describe how the strength of the body of evidence will be assessed (such as GRADE)	Not applicable

From: Shamseer L, Moher D, Clarke M et al; PRISMA-P Group (2015) Preferred reporting items for systematic review and meta-analysis protocols (PRISMA-P) 2015: elaboration and explanation. BMJ 349(jan02 1):g7647

2. PRISMA-S checklist

Section and topic	No	Checklist item	Reported
**INFORMATION SOURCES AND METHODS**			
Database name	1	Name each individual database searched, stating the platform for each.	Method section, Supplementary Note S3
Multi-database searching	2	If databases were searched simultaneously on a single platform, state the name of the platform, listing all of the databases searched.	Method section, Supplementary Note S3
Study registries	3	List any study registries searched.	Method section, Supplementary Note S3
Online resources and browsing	4	Describe any online or print source purposefully searched or browsed (e.g., tables of contents, print conference proceedings, web sites), and how this was done.	Method section, Supplementary Note S3
Citation searching	5	Indicate whether cited references or citing references were examined, and describe any methods used for locating cited/citing references (e.g., browsing reference lists, using a citation index, setting up email alerts for references citing included studies).	Method section, Supplementary Note S3
Contacts	6	Indicate whether additional studies or data were sought by contacting authors, experts, manufacturers, or others.	Not applicable
Other methods	7	Describe any additional information sources or search methods used.	Method section, Supplementary Note S3
**SEARCH STRATEGIES**			
Full search strategies	8	Include the search strategies for each database and information source, copied and pasted exactly as run.	Supplementary Note S3
Limits and restrictions	9	Specify that no limits were used, or describe any limits or restrictions applied to a search (e.g., date or time period, language, study design) and provide justification for their use.	Supplementary Note S3
Search filters	10	Indicate whether published search filters were used (as originally designed or modified), and if so, cite the filter(s) used.	Supplementary Note S3
Prior work	11	Indicate when search strategies from other literature reviews were adapted or reused for a substantive part or all of the search, citing the previous review(s).	Supplementary Note S3
Updates	12	Report the methods used to update the search(es) (e.g., rerunning searches, email alerts).	Not applicable
Dates of searches	13	For each search strategy, provide the date when the last search occurred.	Supplementary Note S3
**PEER REVIEW**			
Peer review	14	Describe any search peer review process.	Supplementary Note S3

MANAGING RECORDS			
Total Records	15	Document the total number of records identified from each database and other information sources.	Figure 1, Supplementary Note S3 and S6
Deduplication	16	Describe the processes and any software used to deduplicate records from multiple database searches and other information sources.	Figure 1, Supplementary Note S3

From: Rethlefsen ML, Kirtley S, Waffenschmidt S et al; PRISMA-S Group (2021) PRISMA-S: an extension to the PRISMA Statement for Reporting Literature Searches in Systematic Reviews. Syst Rev 10(1):39

3. PRISMA 2020 abstract checklist

Section and topic	No	Checklist item	Reported
**TITLE**			
Title	1	Identify the report as a systematic review.	Title
**BACKGROUND**			
Objectives	2	Provide an explicit statement of the main objective(s) or question(s) the review addresses.	Objective
**METHODS**			
Eligibility criteria	3	Specify the inclusion and exclusion criteria for the review.	Methods
Information sources	4	Specify the information sources (e.g. databases, registers) used to identify studies and the date when each was last searched.	Methods
Risk of bias	5	Specify the methods used to assess risk of bias in the included studies.	Methods
Synthesis of results	6	Specify the methods used to present and synthesise results.	Methods
**RESULTS**			
Included studies	7	Give the total number of included studies and participants and summarise relevant characteristics of studies.	Results
Synthesis of results	8	Present results for main outcomes, preferably indicating the number of included studies and participants for each. If meta-analysis was done, report the summary estimate and confidence/credible interval. If comparing groups, indicate the direction of the effect (i.e. which group is favoured).	Results
**DISCUSSION**			
Limitations of evidence	9	Provide a brief summary of the limitations of the evidence included in the review (e.g. study risk of bias, inconsistency and imprecision).	Not reported
Interpretation	10	Provide a general interpretation of the results and important implications.	Conclusion
**OTHER**			
Funding	11	Specify the primary source of funding for the review.	Declaration paragraph
Registration	12	Provide the register name and registration number.	Registration number

From: Page MJ, McKenzie JE, Bossuyt PM et al (2021) The PRISMA 2020 statement: an updated guideline for reporting systematic reviews. BMJ 372:n71

4. PRISMA 2020 checklist

Section and topic	No	Checklist item	Reported
**TITLE**			
Title	1	Identify the report as a systematic review.	Title
**ABSTRACT**			
Abstract	2	See the PRISMA 2020 for Abstracts checklist.	See PRISMA 2020 abstract checklist
**INTRODUCTION**			
Rationale	3	Describe the rationale for the review in the context of existing knowledge.	Introduction section
Objectives	4	Provide an explicit statement of the objective(s) or question(s) the review addresses.	Introduction section
**METHODS**			
Eligibility criteria	5	Specify the inclusion and exclusion criteria for the review and how studies were grouped for the syntheses.	Method section, Supplementary Note S2 and S3
Information sources	6	Specify all databases, registers, websites, organisations, reference lists and other sources searched or consulted to identify studies. Specify the date when each source was last searched or consulted.	Method section, Supplementary Note S2 and S3
Search strategy	7	Present the full search strategies for all databases, registers and websites, including any filters and limits used.	Method section, Supplementary Note S3
Selection process	8	Specify the methods used to decide whether a study met the inclusion criteria of the review, including how many reviewers screened each record and each report retrieved, whether they worked independently, and if applicable, details of automation tools used in the process.	Method section, Supplementary Note S3
Data collection process	9	Specify the methods used to collect data from reports, including how many reviewers collected data from each report, whether they worked independently, any processes for obtaining or confirming data from study investigators, and if applicable, details of automation tools used in the process.	Method section, Supplementary Note S4
Data items	10a	List and define all outcomes for which data were sought. Specify whether all results that were compatible with each outcome domain in each study were sought (e.g. for all measures, time points, analyses), and if not, the methods used to decide which results to collect.	Method section, Supplementary Note S4
	10b	List and define all other variables for which data were sought (e.g. participant and intervention characteristics, funding sources). Describe any assumptions made about any missing or unclear information.	Method section, Supplementary Note S4
Study risk of bias assessment	11	Specify the methods used to assess risk of bias in the included studies, including details of the tool(s) used, how many reviewers assessed each study and whether they worked independently, and if applicable, details of automation tools used in the process.	Method section, Supplementary Note S4
Effect measures	12	Specify for each outcome the effect measure(s) (e.g. risk ratio, mean difference) used in the synthesis or presentation of results.	Method section, Supplementary Note S4

Synthesis methods	13a	Describe the processes used to decide which studies were eligible for each synthesis (e.g. tabulating the study intervention characteristics and comparing against the planned groups for each synthesis (item #5)).	Method section, Supplementary Note S5 and S6
	13b	Describe any methods required to prepare the data for presentation or synthesis, such as handling of missing summary statistics, or data conversions.	Method section, Supplementary Note S5 and S6
	13c	Describe any methods used to tabulate or visually display results of individual studies and syntheses.	Method section, Supplementary Note S5 and S6
	13d	Describe any methods used to synthesize results and provide a rationale for the choice(s). If meta-analysis was performed, describe the model(s), method(s) to identify the presence and extent of statistical heterogeneity, and software package(s) used.	Not applicable
	13e	Describe any methods used to explore possible causes of heterogeneity among study results (e.g. subgroup analysis, meta-regression).	Not applicable
	13f	Describe any sensitivity analyses conducted to assess robustness of the synthesized results.	Not applicable
Reporting bias assessment	14	Describe any methods used to assess risk of bias due to missing results in a synthesis (arising from reporting biases).	Method section, Supplementary Note S5 and S6
Certainty assessment	15	Describe any methods used to assess certainty (or confidence) in the body of evidence for an outcome.	Not applicable
**RESULTS**			
Study selection	16a	Describe the results of the search and selection process, from the number of records identified in the search to the number of studies included in the review, ideally using a flow diagram.	Results section, Figure 1, Table 1
	16b	Cite studies that might appear to meet the inclusion criteria, but which were excluded, and explain why they were excluded.	Results section, Supplementary Note S6
Study characteristics	17	Cite each included study and present its characteristics.	Results section
Risk of bias in studies	18	Present assessments of risk of bias for each included study.	Results section, Figure 2, Table 2
Results of individual studies	19	For all outcomes, present, for each study: (a) summary statistics for each group (where appropriate) and (b) an effect estimate and its precision (e.g. confidence/credible interval), ideally using structured tables or plots.	Results section, Figure 2, Table 2
Results of syntheses	20a	For each synthesis, briefly summarise the characteristics and risk of bias among contributing studies.	Results section, Figure 3, Table 3
	20b	Present results of all statistical syntheses conducted. If meta-analysis was done, present for each the summary estimate and its precision (e.g. confidence/credible interval) and measures of statistical heterogeneity. If comparing groups, describe the direction of the effect.	Not applicable
	20c	Present results of all investigations of possible causes of heterogeneity among study results.	Not applicable

	20d	Present results of all sensitivity analyses conducted to assess the robustness of the synthesized results.	Not applicable
Reporting biases	21	Present assessments of risk of bias due to missing results (arising from reporting biases) for each synthesis assessed.	Results section, Figure 2, Table 2
Certainty of evidence	22	Present assessments of certainty (or confidence) in the body of evidence for each outcome assessed.	Not applicable
**DISCUSSION**			
Discussion	23a	Provide a general interpretation of the results in the context of other evidence.	Discussion section
	23b	Discuss any limitations of the evidence included in the review.	Discussion section
	23c	Discuss any limitations of the review processes used.	Discussion section
	23d	Discuss implications of the results for practice, policy, and future research.	Discussion section
**OTHER INFORMATION**			
Registration and protocol	24a	Provide registration information for the review, including register name and registration number, or state that the review was not registered.	Abstract, Method section
	24b	Indicate where the review protocol can be accessed, or state that a protocol was not prepared.	Supplementary Note S2
	24c	Describe and explain any amendments to information provided at registration or in the protocol.	Supplementary Note S2
Support	25	Describe sources of financial or non-financial support for the review, and the role of the funders or sponsors in the review.	Declaration paragraph
Competing interests	26	Declare any competing interests of review authors.	Declaration paragraph
Availability of data, code and other materials	27	Report which of the following are publicly available and where they can be found: template data collection forms; data extracted from included studies; data used for all analyses; analytic code; any other materials used in the review.	Supplementary Materials

From: Page MJ, McKenzie JE, Bossuyt PM et al (2021) The PRISMA 2020 statement: an updated guideline for reporting systematic reviews. BMJ 372:n71

Supplementary Note S2 Review protocol

PROSPERO registration
First drafted date: 30 June 2023
PROSEPRO submitted date: 01 July 2023
Last edited date: 01 July 2023
Temporary ID: 441532
PROSEPRO ID: CRD42023441532

Review title
Appraisal of guidelines on intravenous contrast media use in patients with kidney disease

Review question
(1) To systematically evaluate the quality of guidelines on intravenous contrast media use in patients with kidney disease; (2) to highlight consistencies of the recommendations to inform the best practice, and (3) to identify the disagreements among guidelines for consideration in future investigations.

Searches
Literature search will be conducted in six peer-reviewed electronic databases (PubMed, Embase, Web of Science, Cochrane Library, China National Knowledge Infrastructure, Wanfang Data), and eight guideline libraries (Guidelines International Network library of guidelines, World Health Organization guidelines, National Institute for Health and Care Excellence, Scottish Intercollegiate Guidelines Network, Canadian Medical Association clinical practice guideline Infobase, New Zealand Guidelines Group, Chinese Medical Ace Base, Practice guideline REgistration for transPAREncy) from 01 January 2018 to present for guidelines on contrast media use in patients with kidney disease. We restricted the publication time from 01 January 2018 onwards to present the recent developments on this topic, and only guidelines written in English and Chinese were available. We will screen the reference lists of all included guidelines and consult the experts for potentially eligible guidelines that are not indexed in the aforementioned databases.

[Modification]
To identify potential eligible guidelines, we will further screen ten homepages of radiological societies (International Society of Radiology, European Society of Radiology, Radiological Society of North America, American Roentgen Ray Society, American College of Radiology, Canadian Association of Radiologists, The Royal College of Radiologists, The Royal Australian and New Zealand College of Radiologists, Japan Radiological Society, Chinese Society of Radiology). The homepages of radiological societies may provide relevant guidelines endorsed by these professional societies.

Condition or domain being studied
Diagnostic imaging with intravenous contrast media has been widely used in the clinical practice and provides a large amount of valuable information. The high safety profile guarantees the use of millions of doses of modern contrast media worldwide. However, due to the concern on the post-contrast acute kidney injury (PC-AKI), intravenous contrast media have been historically denied or delayed in patients with kidney diseases. Unnecessary delay in diagnostic imaging brings the potential for indirect harm due to delayed diagnosis or misdiagnosis. It would be necessary to summarize the current guidelines to aid the radiologists and clinicians in balancing the trade-off between the potential risks of contrast media and diagnostic benefits.

Participants/population
We will include the lasted versions of clinical practice guidelines published on intravenous contrast media use in patients with kidney disease. The following articles were excluded: (1) guidelines developed from the perspective of a medical specialty, in which contrast media were discussed as one of the many risk factors for kidney injury; (2) guidelines on intra-arterial contrast media administration, because intra-arterial administration has unique considerations that do not apply to the intravenous route of administration; (3) a previous version of an updated guidelines or a guideline under development; (4) study protocols, primary studies, comments on guidelines, conference abstracts, or other not guidance documents; (5) duplications.

Intervention(s), exposure(s)
Not applicable.

Comparator(s)/control
Not applicable.

Types of study to be included
The types of study to be included are limited to clinical practice guidelines. The guidelines were defined as document that identified itself as a guideline, or a guidance document with recommendations including consensus, appropriateness criteria, manual, etc. The disagreements were resolved by discussion or consults within the review group.

Main outcome(s)
Quality appraisal of included clinical practice guidelines on intravenous contrast media use in patients with kidney disease.

Measures of effect
Quality scores according to the Scientific, Transparent and Applicable Rankings (STAR) tool.

Additional outcome(s)
Summary of recommendations on intravenous contrast media use in patients with kidney disease.

Measures of effect
A descriptive summary of guideline recommendations.

Data extraction (selection and coding)
Two independent reviewers will be extracted the data from all available materials of each guideline according to predefined data extraction tool. This tool includes bibliographical information, characteristics, development details, and key recommendations. The disagreements were resolved by discussion or consults with a third independent reviewer.

Risk of bias (quality) assessment
Two independent reviewers will evaluate the quality of included guidelines by using the Scientific, Transparent and Applicable Rankings (STAR) tool. The STAR guideline evaluation tool has good reliability and validity, and clear advantages in efficiency compared to existing tools. This tool is therefore well suited for the comprehensive evaluation of clinical practice guidelines.

Strategy for data synthesis
The STAR score will be calculated. The key recommendations from the included guidelines will be qualitative summarized.

Analysis of subgroups or subsets
Not planned.

Type and method of review
Type of review: Methodology; Narrative synthesis; Systematic review
Health area of the review: Urological

Keywords
Contrast media; Kidney disease

Conflicts of interest
None.

Funding
This study has received funding by National Natural Science Foundation of China (82302183, 82271934); Yangfan Project of Science and Technology Commission of Shanghai Municipality (22YF1442400); and Research Fund of Tongren Hospital,

Shanghai Jiao Tong University School of Medicine (TRKYRC-XX202204, TRYJ2021JC06, TRGG202101). They played no role in the study design, data collection or analysis, decision to publish, or manuscript preparation.

Supplementary Note S3 Search strategy and study selection

Search strategy
Our review group is consisted of experts with various professional backgrounds including: radiologists (JYZ, YX, YFH, DFD, XG, LJL, HZ, WWY), radiographers (LWC, WJL, SLW, YZ), a nephrologist (YPS), a urologist (YBW), a statistician (JJL), a pharmacist (YD), and an expert in pharmacovigilance (RJ). The selection of the information sources was based on the previous studies and experts' opinions. The search strings were decided according to a preliminary search.

Discussions on the information source
(1) The previous similar studies (Eur Radiol, https://doi.org/10.1007/s00330-023-09786-8; Eur Radiol, https://doi.org/10.1007/s00330-023-09611-2) included only PubMed and Embase, but we include more peer-reviewed literature databases: PubMed, Embase, Web of Science, Cochrane Library, China National Knowledge Infrastructure, Wanfang Data. In addition to PubMed and Embase, we believe the Web of Science should also be include as an information source since it is another trustworthy database for potential guidelines. The Cochrane Library is an important information source for evidence-based reviews. Further, benefited by the diversity of our review group, we were able to include and assess the literatures in Chinese from China National Knowledge Infrastructure, and Wanfang Data.

(2) The previous similar studies (Eur Radiol, https://doi.org/10.1007/s00330-023-09786-8; Eur Radiol, https://doi.org/10.1007/s00330-023-09611-2) included guideline libraries such as National Guidelines Clearing House, ECRI Guidelines Trust, National Health Service Evidence and the Scottish Intercollegiate Guidelines Network. Our study included more guideline libraries to allow a more complete search. The included guideline libraries are Guidelines International Network library of guidelines, World Health Organization guidelines, National Institute for Health and Care Excellence, Scottish Intercollegiate Guidelines Network, Canadian Medical Association clinical practice guideline Infobase, New Zealand Guidelines Group, Chinese Medical Ace Base, Practice guideline REgistration for transPAREncy. We believe the extended scope of searching can make our study more reasonable.

(3) The previous similar studies (Eur Radiol, https://doi.org/10.1007/s00330-023-09786-8; Eur Radiol, https://doi.org/10.1007/s00330-023-09611-2) included homepages of radiological societies such as European Society of Radiology, The Royal Australian and New Zealand College of Radiologists, American College of Radiology, and Japan Radiological Society. Although there were more societies mentioned in the previous studies, we did not include those unrelated to our study such as nuclear medicine societies and diabetes societies. In addition to the radiological societies mentioned in the previous studies. We further included websites of International Society of Radiology, Radiological Society of North America, American Roentgen Ray Society, Canadian Association of Radiologists, The Royal College of Radiologists, and Chinese Society of Radiology, for more potential guidelines.

According to to the recommendations in the PRISMA Statement for Reporting Literature Searches in Systematic Reviews (Syst Rev, 2021, 10(1):39). As in the statement: "There is no single database that is able to provide a complete and accurate list of all studies that meet systematic review criteria due to the differences in the articles included and the indexing methods used between databases. These differences have led to recommendations that systematic review teams search multiple databases to maximize the likelihood of finding relevant studies. This may include using broad disciplinary databases, specialized databases, or regional databases." Therefore, we believe our search that covers a wider range of information sources than previous studies can better avoid the bias related to the databases, and present the current status of the guideline on intravenous iodinated contrast media use in patients with kidney disease.

Development of search string
We firstly performed a preliminary search to confirm the availability of the search string. One of the reviewers (JYZ) has experience in developing the search strings. The reviewer developed the search stirrings and validated their feasibility. The search string was developed by combining the variations of the terms of "contrast media", "kidney", and either "guideline", "consensus", "statement", etc. Then, we conducted the formal search to identify potential available articles. The literature search was duplicated by two independent reviewers (JYZ and YX). The disagreements were resolved by

consults with the review group. Our review group is consisted of experts with varies professional backgrounds including radiologists (JYZ, YX, YFH, DFD, XG, LJL, HZ, WWY), radiographers (LWC, WJL, SLW, YZ), a nephrologist (YPS), a urologist (YBW), a statistician (JJL), a pharmacist (YD), and an expert in pharmacovigilance (RJ).

Formal literature search

Literature search will be conducted in six peer-reviewed electronic databases (PubMed, Embase, Web of Science, Cochrane Library, China National Knowledge Infrastructure, Wanfang Data), eight guideline libraries (Guidelines International Network library of guidelines, World Health Organization guidelines, National Institute for Health and Care Excellence, Scottish Intercollegiate Guidelines Network, Canadian Medical Association clinical practice guideline Infobase, New Zealand Guidelines Group, Chinese Medical Ace Base, Practice guideline REgistration for transPAREncy), and ten homepages of radiological societies (International Society of Radiology, European Society of Radiology, Radiological Society of North America, American Roentgen Ray Society, American College of Radiology, Canadian Association of Radiologists, The Royal College of Radiologists, The Royal Australian and New Zealand College of Radiologists, Japan Radiological Society, Chinese Society of Radiology) from 01 January 2018 to present for guidelines on contrast media use in patients with kidney disease. We restricted the publication time from 01 January 2018 onwards to present the recent developments on this topic, and only guidelines written in English and Chinese were available. We will screen the reference lists of all included guidelines and consult the experts for potentially eligible guidelines that are not indexed in the aforementioned databases. The additionally eligible guidelines were distinguished by screening the reference lists of all included guidelines and consulting experts.

1. Peer-reviewed databases

1.1 PubMed

Available via https://pubmed.ncbi.nlm.nih.gov
Preliminary search date: 01 Jul 2023
Articles retrieved: 275
Publication time: 2018-2023 ((2018:2023[pdat]))
Search string: (("contrast"[Title/Abstract]) AND ("kidney"[Title/Abstract] OR "renal"[Title/Abstract] OR "nephr*"[Title/Abstract]) AND ("guideline"[Title/Abstract] OR "consensus"[Title/Abstract] OR "statement"[Title/Abstract] OR "recommendation"[Title/Abstract]))

1.2 Embase

Available via www.embase.com
Preliminary search date: 01 Jul 2023
Articles retrieved: 421
Publication time: 2018-2023 ((2018:py OR 2019:py OR 2020:py OR 2021:py OR 2022:py OR 2023:py))
Search string: ("contrast":ti,ab,kw) AND ("kidney":ti,ab,kw OR " renal":ti,ab,kw OR " nephr*":ti,ab,kw) AND ("guideline":ti,ab,kw OR "consensus":ti,ab,kw OR "statement":ti,ab,kw)

1.3 Web of Science

Available via apps.webofknowledge.com
Preliminary search date: 01 Jul 2023
Articles retrieved: 1123
Publication time: 2018-2023 (PY=(2018 OR 2019 OR 2020 OR 2021 OR 2022 OR 2023))
Search string: (TS=(contrast)) AND (TS=(kidney) OR TS=(renal) OR TS=(nephr*)) AND (TS=(guideline) OR TS=(consensus) OR TS=(statement) OR TS=(recommendation))

1.4 Cochrane review via Cochrane Library

Available via https://www.cochranelibrary.com
Preliminary search date: 01 Jul 2023
Articles retrieved: 528 (Cochrane Reviews 351, Cochrane Protocol 55, Trials 122)
Publication time: 2018-2023 (with Cochrane Library publication date from Jan 2018 to Dec 2023)
Search string: contrast AND (kidney OR renal OR nephr*) AND (guideline OR consensus OR statement OR recommendation)

1.5 China National Knowledge Infrastructure
Available via http://www.cnki.net
Preliminary search date: 01 Jul 2023
Articles retrieved: 99
Publication time: 2018-2023
Search string: (TKA="造影剂" OR TKA="对比剂") AND (TKA="指南" OR TKA="共识")
English translation: (contrast) AND (guideline OR consensus)

1.6 Wanfang Data
Available via https://www.wanfangdata.com.cn
Preliminary search date: 01 Jul 2023
Articles retrieved: 115
Publication time: 2018-2023
Search string: (主题:("造影剂") OR 主题:("对比剂")) AND (主题:("指南") OR 主题:("共识"))
English translation: (contrast) AND (guideline OR consensus)

2. Guideline libraries
2.1 Guidelines International Network library of guidelines
Available via https://g-i-n.net/international-guidelines-library/ OR https://guidelines.ebmportal.com/
Preliminary search date: 01 Jul 2023
Articles retrieved: 0
Search string: contrast

2.2 World Health Organization guidelines
Available via https://www.who.int/publications/who-guidelines
Preliminary search date: 01 Jul 2023
Articles retrieved: 0
Search string: contrast

2.3 National Institute for Health and Care Excellence
Available via http://guidance.nice.org.uk
Preliminary search date: 01 Jul 2023
Articles retrieved: 125
Search string: contrast

2.4 Scottish Intercollegiate Guidelines Network
Available via http://www.sign.ac.uk
Preliminary search date: 01 Jul 2023
Articles retrieved: 1
Search string: contrast

2.5 Canadian Medical Association clinical practice guideline Infobase
Available via https://joulecma.ca/cpg/homepage
Preliminary search date: 01 Jul 2023
Articles retrieved: 4
Search string: contrast

2.6 New Zealand Guidelines Group
Available via https://www.health.govt.nz/publications
Preliminary search date: 01 Jul 2023
Articles retrieved: 11
Search string: contrast

2.7 Chinese Medical Ace Base
Available via http://seleguide.yiigle.com/home/zhinan
Preliminary search date: 01 Jul 2023
Articles retrieved: 11
Search string: "造影剂", "对比剂"
English translation: contrast

2.8 Practice guideline REgistration for transPAREncy
Available via www.guidelines-registry.cn
Preliminary search date: 01 Jul 2023
Articles retrieved: 0
Search string: "造影剂", "对比剂"
English translation: contrast

3. Homepages of Radiological Societies
3.1 International Society of Radiology
Available via http://isradiology.org
Preliminary search date: 01 Jul 2023
Articles retrieved: 2
Search string: contrast

3.2 European Society of Radiology
Available via https://www.myesr.org
Preliminary search date: 01 Jul 2023
Articles retrieved: 14 (14 published 2018-2023 on Guidelines & Recommendations page)

3.3 Radiological Society of North America
Available via https://www.rsna.org/
Preliminary search date: 01 Jul 2023
Articles retrieved: 2
Search string: contrast[title]

3.4 American Roentgen Ray Society
Available via https://arrs.org
Preliminary search date: 01 Jul 2023
Articles retrieved: 0 (Related materials not found)

3.5 American College of Radiology
Available via https://www.acr.org
Preliminary search date: 01 Jul 2023
Articles retrieved: 1 (Contrast Manual on the Contrast Manual page)

3.6 Canadian Association of Radiologists
Available via https://www.car.ca
Preliminary search date: 01 Jul 2023
Articles retrieved: 14 (14 published 2018-2023 on Practice Guidelines page, 3 were contrast-related)

3.7 Royal College of Radiologists
Available via https://www.rcr.ac.uk
Preliminary search date: 01 Jul 2023
Articles retrieved: 50
Search string: contrast

3.8 Royal Australian and New Zealand College of Radiologists
Available via https://www.ranzcr.com
Preliminary search date: 01 Jul 2023
Articles retrieved: 20
Search string: contrast

3.9 Japan Radiological Society
Available via http://www.radiology.jp
Preliminary search date: 01 Jul 2023
Articles retrieved: 1 (1 related found on guideline page)

3.10 Chinese Society of Radiology
Available via https://csr.cma.org.cn/cn/
Preliminary search date: 01 Jul 2023
Articles retrieved: 1 (1 related found on guideline page)

Study selection
The titles and abstracts of unique records were screened by two independent reviewers (JYZ and YX), and then their eligibility was confirmed by reading the full-texts and supplementary materials. The supplementary materials including but limited to protocol, conflict of interest declaration, evidence summary, and dissemination materials. For the guidelines published on multiple journals, all available materials were evaluated as a whole. The disagreements were resolved by discussion or consults with the review group. Our review group is consisted of experts with varies professional backgrounds including radiologists (JYZ, YX, YFH, DFD, XG, LJL, HZ, WWY), radiographers (LWC, WJL, SLW, YZ), a nephrologist (YPS), a urologist (YBW), a statistician (JJL), a pharmacist (YD), and an expert in pharmacovigilance (RJ).

The following articles were included:
(1) guidelines on iodinated contrast medium in patients with kidney disease, which were defined as document that identified itself as a guideline, or a guidance document with recommendations including consensus, appropriateness criteria, manual, etc.;
(2) guidelines are reported in English, or Chinese;
(3) the publication time from 1 January 2018 onwards to present;
(4) institutional full-text availability.

The following articles were excluded:
(1) guidelines developed from the perspective of a medical specialty, in which contrast media were discussed as one of the many risk factors for kidney injury;
(2) guidelines on intra-arterial contrast media administration, because intra-arterial administration has unique considerations that do not apply to the intravenous route of administration;
(3) a previous version of an updated guidelines or a guideline under development;
(4) study protocols, primary studies, comments on guidelines, conference abstracts, or other not guidance documents;
(5) duplications.

We did not consider that the guidelines or consensus should be published as a scientific article. For example, the manual on contrast from American College of Radiology is only available on the website of the American College of Radiology (https://www.acr.org/Clinical-Resources/Contrast-Manual). However, this does not hinder the importance of this manual, and the application of this manual in the clinical practice. Further, the length of a guideline or a consensus paper may exceed the limitation of a journal. For example, the guideline of The Royal Australian and New Zealand College of Radiologists (https://www.ranzcr.com/search/ranzcr-iodinated-contrast-guidelines) is too long to published as an article, as well as the full version of the guideline of European Society of Urogenital Radiology (https://www.esur.org/wp-content/uploads/2022/03/ESUR-Guidelines-10_0-Final-Version.pdf). It is not possible to require all the guidelines should be published as a scientific article. The guidelines endorsed by the radiological societies should be as recognized as guidelines.

Supplementary Note S4 Data extraction and quality appraisal

Data extraction

We developed a data extraction sheet to collect study data. As the reviewers have different levels of experience and knowledge, the items listed were reviewed and discussed to ensure that all reviewers had clear knowledge of the procedures. Due to there were only several guidelines included in this study, the training phase was not introduced before the formal extraction. The three independent reviewers (JYZ and either LWC or YX) directly conducted the data extraction. The disagreements were resolved by consults with the review group. Our review group is consisted of experts with varies professional backgrounds including radiologists (JYZ, YX, YFH, DFD, XG, LJL, HZ, WWY), radiographers (LWC, WJL, SLW, YZ), a nephrologist (YPS), a urologist (YBW), a statistician (JJL), a pharmacist (YD), and an expert in pharmacovigilance (RJ).

The following items were discussed to reach consensus:

(1) The bibliographical information (guideline name, journal name, publication year, citation information) was collected according to the Web of Science (apps.webofknowledge.com).
(2) The region of the guideline was decided according to the author or organization of the guideline.
(3) The nine questions for identifying the key recommendations were generated by discussion among reviewers. The radiologists (ZJY and YX) with 5- to 6-years of experience in CT image interpretation, and radiographers (LWC and YZ) with 15- to 25-years of experience in X-ray and CT acquisition lead the discussion. The two most experienced radiologists (WWY and HZ) with 28- and 30-years of experience in image interpretation supervised the discussion.

Quality appraisal

We evaluated the quality of included guidelines by using the Scientific, Transparent and Applicable Rankings (STAR) tool. As the reviewers have different levels of experience and knowledge, the items listed were reviewed and discussed to ensure that all reviewers had clear knowledge of the procedures. Due to there were only several guidelines included in this study, the training phase was not introduced before the formal extraction. The three independent reviewers (JYZ and either LWC or YX) directly conducted the quality appraisal. The disagreements were resolved by consults with the review group. Our review group is consisted of experts with varies professional backgrounds including radiologists (JYZ, YX, YFH, DFD, XG, LJL, HZ, WWY), radiographers (LWC, WJL, SLW, YZ), a nephrologist (YPS), a urologist (YBW), a statistician (JJL), a pharmacist (YD), and an expert in pharmacovigilance (RJ).

Discussions on choosing the STAR tool for quality appraisal

(1) The STAR checklist does include aspects that is not assessed by other international guidelines such as RIGHT or AGREE-II. For example, the first domain "Registry" is not mentioned in neither RIGHT nor AGREE-II. Take another example, the second domain "Protocol" is also missing in both RIGHT and AGREE-II. It is surprising for us that the RIGHT and AGREE-II did not mention the registry and protocol for guideline development which is the fundamental for the methodological robustness. Therefore, we believe there is some aspect that is not assessed by other international guidelines.
(2) It is true that the STAR checklist is firstly applied to evaluate the quality of Chinese guidelines. However, it is not only suitable for the Chinese guidelines. In the original STAR paper, the STAR tool is developed intended for use by a wide range of users, including healthcare providers, policymakers, and guideline methodologists and researchers. There is no region limitation for the tool that only can be used in China.
(3) There are external validations of the STAR tool. After the development of the STAR tool, the Guidelines and Standards Research Center Chinese Medical Association Publishing House, Research Unit of Evidence-Based Evaluation and Guidelines, WHO Collaborating Centre for Guideline Implementation and Knowledge Translation, and the Scientific, Transparent and Applicable Rankings (STAR) Working Group have already organized the evaluation of the guidelines using STAR tool on 2021 and 2022. The group evaluated 291 guidelines and 858 consensuses on 2021 (Evaluation and ranking for scientific, transparent and applicable of Chinese guidelines and consensus published in the medical journals in 2021, https://doi.org/10.3760/cma.j.cn112137-20220602-01232 [Article in Chinese]), and 334 guidelines and 1143 consensuses on 2022 (Evaluation and ranking for scientific, transparent and applicable of Chinese guidelines and consensus published in the medical journals in 2022, https://doi.org/10.3760/cma.j.cn112137-20230724-00076 [Article in Chinese]) using STAR tool. We believe these applications of STAR tool can well validate the tool.

The following items were discussed to reach consensus:

(1) **Items 5-7:** If the guideline declare that it has not receive any funding (item 5), it would be rated as 1. Then the items 6 and 7 are rated as 1, since it is not expectable that the guideline would report the role and influence on the guideline when there was no funding. It is reasonable to presume that the funding would not play a role in or influence on the guideline when there was no funding.

(2) **Items 11-12:** If there were experts from different disciplines (e.g., from department of, nephrology, medical oncology, surgery, etc.), the expert would be considered as experts from at least two disciplines in addition to the guideline's topic who took part in the development. However, item 11 would be rated as 0.5, since the item was not clearly declared. A librarian is considered as a methodologist. However, item 12 would be rated as 0.5, since the item was not clearly declared.

(3) **Items 13-14:** If the guideline declare that there were no conflicts of interest (item 13), it would be rated as 1. Then the item 14 is rated as 1, since it is not expectable that the guideline would report the evaluation and management of conflicts of interest when there were conflicts of interest. It is reasonable to presume that the conflicts of interest would not influence on the guideline when there were conflicts of interest.

(4) **Item 35:** If the guideline can be found in the database, guideline libraries, or the homepages of radiological societies, the item would be rated as 0.5. If the guideline were published on two or more than two journals, the item would be rated as 0.5.

(5) **Item 38:** If the guideline can be download without institutional availability, then this item would be rated as 1. Or it would be rated as 0.

Supplementary Note S5 Data analysis process

Statistical analysis

The statistical analysis was performed with R language version 4.1.3 within RStudio version 3.6.3 by using relevant packages. The data analysis was performed by a reviewer (JYZ) under supervision of a statistical expert (JJL). However, there are no complex statistical analysis necessary for this study. The sum STAR score can be easily calculated as the sum of domain weight × item weight × item score of 37 items, with a maximum sum score of 100.

Summary of the key recommendations

The reviewer group is aimed to compare the key recommendations and summarize the (1) the consistencies of the recommendations to inform the best practice, and (2) to identify the disagreements among guidelines for consideration in future investigations. Before the first discussion among the reviewers, three reviewers (JYZ and LWC, YX) firstly identified the key recommendations according for (1) screening patients with regard to their need for renal function testing, (2) clinical estimation of renal function, (3) contraindications for risk stratification of PC-AKI, (4) peri-administration strategies to mitigate the risk of PC-AKI in higher risk individuals. The key recommendations were summarized as Supplementary Table S4.

During the first discussion among the reviewers, the reviewer group read the key recommendations for discussion, and listed the following questions for further evaluation. (1) Who should undergo renal function test before contrast media administration? (2) How long is the time interval between renal function test and administration acceptable? (3) What is the eGFR cutoff for patients at risk of acute kidney injury/ needs referring/ further treatment? (4) What kind of contrast media is recommended for high-risk patients? (5) Is reduced contrast media dosage recommended for high-risk patients? (6) How long is the suitable time interval between scan and repeated scan? (7) Is hydration recommended for high-risk patients, and how? (8) Is any drug recommended for high-risk patients, and what are they? (9) Is blood purification therapy recommended for high-risk patients, and how? After the first discussion Three reviewers (JYZ, LWC, and YX) summarized the answers before the second discussion and summarized as Supplementary Table S5.

During the second discussion among the reviewers, the reviewer group discussed the summarized answers for the interested questions. The reviewers discussed and determined whether/ what the guideline recommended/ not recommended/ no comment on each answer for the questions. Three reviewers (JYZ, LWC, and YX) simplified the interested questions and corresponding answers for further discussion.

Table 3 and Figure 3 present a summary for the interested questions and corresponding answers were summarized according to the second discussion. All the reviewers read and approved the final version of the summary of recommendations.

Supplementary Note S6 Excluded records of full-texts with justifications

1. Krestan C (2019) Kontrastmittel – Handlungsempfehlungen für die Praxis [Contrast media - Guidelines for practical use]. Radiologe 59(5):444-453. **(in German)**
2. Pein U, Fritz A, Markau S, Wohlgemuth WA, Girndt M (2021) Kontrastmittelgabe bei Niereninsuffizienz – praktische Handlungsempfehlungen [Contrast media use in kidney disease - clinical practice recommendations]. Dtsch Med Wochenschr 146(22):1489-1495 **(in German)**
3. Hinson JS, Ehmann MR, Klein EY (2020) Evidence and Patient Safety Prevail Over Myth and Dogma: Consensus Guidelines on the Use of Intravenous Contrast Media. Ann Emerg Med 76(2):149-152 **(Comment or summary of guideline)**
4. Maas M, Beets-Tan R, Gaubert JY et al (2020) Follow-up after radiological intervention in oncology: ECIO-ESOI evidence and consensus-based recommendations for clinical practice. Insights Imaging 11(1):83 **(Not focus on contrast)**
5. de Laforcade L, Bobot M, Bellin MF et al (2021) Kidney and contrast media: Common viewpoint of the French Nephrology societies (SFNDT, FIRN, CJN) and the French Radiological Society (SFR) following ESUR guidelines. Diagn Interv Imaging 102(3):131-139 **(Comment or summary of guideline)**
6. Nijssen EC, Rennenberg R, Nelemans P, van Ommen V, Wildberger JE (2021) Post-Contrast Acute Kidney Injury and Intravenous Prophylactic Hydration: An Update. Rofo 193(2):151-159 **(in German)**
7. Sebastià C, Nicolau C, Martín de Francisco ÁL, Poch E, Oleaga L (2020) Prophylaxis against postcontrast acute kidney injury (PC-AKI): updates in the ESUR guidelines 10.0 and critical review. Radiologia (Engl Ed) 62(4):292-297 **(Comment or summary of guideline)**
8. Nyman U, Brismar T, Carlqvist J et al (2023) Revised Swedish guidelines on intravenous iodine contrast medium-induced acute kidney injury 2022: A summary. Acta Radiol 64(5):1859-1864 **(Comment or summary of guideline)**
9. Chen JJ, Kuo G, Hung CC et al (2021) Risk factors and prognosis assessment for acute kidney injury: The 2020 consensus of the Taiwan AKI Task Force. J Formos Med Assoc 120(7):1424-1433 **(Not focus on contrast)**
10. Nijssen EC, Nelemans PJ, Rennenberg RJ, van Ommen V, Wildberger JE (2018) Evaluation of Safety Guidelines on the Use of Iodinated Contrast Material: Conundrum Continued. Invest Radiol 53(10):616-622 **(Comment or summary of guideline)**
11. 中国抗癌协会肿瘤影像专业委员会 (2022) 恶性肿瘤患者 CT 增强扫描对比剂安全管理专家共识（2022）. 中华放射学杂志 56(9):941-949 [English translation: Oncology Imaging Professional Committee of China Anti-Cancer Association (2022) Expert consensus on the safety management of CT-enhanced scanning in patients with malignancy (2022). Chinese Journal of Radiology 56(9):941-949] **(Not related to kidney)**
12. 中华护理学会内科专业委员会 (2021) 含碘对比剂静脉外渗护理管理实践指南. 中华护理杂志 56(7):1008 [English translation: Internal Medicine Nursing Committee of Chinese Nursing Association (2021) Clinical practice guideline for nursing management of iodinated contrast media extravasation. Chinese Journal of Nursing 56(7):1008] **(Not related to kidney)**
13. 中华医学会放射学分会放射护理专业委员会放射诊断护理学组 (2018) 影像科碘对比剂输注安全专家共识. 介入放射学杂志 27(8):707-712 [English translation: Diagnostic Nursing Group of Professional Committee on Radiological Nursing Care of Radiology Branch of Chinese Medical Association (2018) Expert consensus on the safety of iodine contrast agent infusion in imaging department Radiological. Journal of Interventional Radiology 27(8):707-712] **(Not related to kidney)**
14. Stacul F, Bertolotto M, Thomsen HS et al; ESUR Contrast Media Safety Committee (2018) Iodine-based contrast media, multiple myeloma and monoclonal gammopathies: literature review and ESUR Contrast Media Safety Committee guidelines. Eur Radiol 28(2):683-691 **(Not related to kidney)**
15. National Institute for Health and Care Excellence (2019) Acute kidney injury: prevention, detection and management Available via https://www.nice.org.uk/guidance/ng148 Accessed 15 July 2023 **(Not focus on contrast)**
16. National Institute for Health and Care Excellence (2019) Point-of-care creatinine devices to assess kidney function before CT imaging with intravenous contrast. Available via https://www.nice.org.uk/guidance/dg37 Accessed 15 July 2023 **(Not focus on contrast)**

Supplementary Table S1 data extraction tool

Domain	Items
Bibliographical information	Name of the guideline
	Author/ organization of the guideline
	Journal published
	Year published
	Language published
	Citation information
	Region published
Characteristics	Registry
	Protocol
	Funding
	Guideline development groups
	Conflicts of interest
	Clinical questions
	Evidence
	Consensus method
	Accessibility
Key recommendations	Who should undergo renal function test before contrast media administration?
	How long is the time interval between renal function test and administration acceptable?
	What is the eGFR cutoff for patients at risk of acute kidney injury/ needs referring/ further treatment?
	What kind of contrast media is recommended for high-risk patients?
	Is reduced contrast media dosage recommended for high-risk patients?
	How long is the suitable time interval between scan and repeated scan?
	Is hydration recommended for high-risk patients, and how?
	Is any drug recommended for high-risk patients, and what are they?
	Is blood purification therapy recommended for high-risk patients, and how?

Supplementary Table S2 STAR tool checklist

Domain	Domain weight	Item	Item wight	Item score
Registry	0.050	1. Register the guideline on an appropriate platform.	0.293	1.5
		2. Provide information about the registry platform and registry ID of the guideline.	0.707	3.5
Protocol	0.050	3. Provide details of the guideline protocol.	0.377	1.9
		4. Identify how the guideline protocol is accessible from an open-source platform<br>(e.g., guideline registry platform or website).	0.623	3.1
Funding	0.031	5. Describe the sources of funding for the development of the guideline.	0.305	1.0
		6. Describe the role of funder(s) in the guideline development.	0.289	0.9
		7. Declare that the funder(s) did not influence the guideline's recommendations.	0.406	1.3
Guideline development groups	0.073	8. List the institutional affiliations of all individuals involved in developing the guideline.	0.128	0.9
		9. Describe the composition of the development groups.	0.137	1.0
		10. Describe the responsibilities of all individuals or sub-groups involved in developing the guideline.	0.175	1.3
		11. Identify experts from at least two disciplines in addition to the guideline's topic who took part in the development.	0.182	1.3
		12. Identify guideline methodologists or experts in evidence-based medicine who took part in the development.	0.378	2.8
Conflicts of interest	0.092	13. Describe whether conflicts of interest existed.	0.474	4.4
		14. Indicate information about the evaluation and management of conflicts of interest.	0.526	4.8
Clinical questions	0.170	15. Identify the clinical questions that the guideline focuses on.	0.377	6.4
		16. Introduce the methods of collecting clinical questions, such as literature search, survey of users, or consultation of experts.	0.146	2.5
Evidence	0.170	17. Indicate how the clinical questions were selected and sorted.	0.197	3.4
		18. Format clinical questions in PICO (population/patients, intervention, control/comparator, and outcome) or other formats.	0.281	4.8
		19. Identify the references for evidence supporting the main recommendations.	0.098	1.7
		20. State to the details of the systematic search (e.g., names of databases, selection criteria, search strategies).	0.131	2.2
		21. Indicate the inclusion and exclusion criteria of research evidence.		1.5
		22. Assess the risk of bias or methodological quality of the included studies.	0.090	1.9
		23. Summarize and analyze the research evidence.	0.113	2.1
		24. Indicate the standard used to grade the evidence quality.	0.125	2.2
		25. Provide the GRADE evidence profile or summary of the results of evidence grading.	0.132	2.4
		26. Provide reference to the full text of systematic reviews.	0.139	1.7
		27. Identify the clinical questions with insufficient evidence (low quality) and indicate future research directions to collect more evidence.	0.101	1.2

Consensus method	0.107	28. Indicate the specific method(s) used to reach consensus (e. g., the Delphi method, Nominal group technique, or informal approaches).	0.072	5.1
		29. Describe the criteria to inform decisions other than the certainty of the evidence (e.g., resource requirements, preferences and values of patients, cost–benefit balance, accessibility, health equity, acceptability). 30. Provide the records of the consensus process.	0.478	3.8
		30. Provide the records of the consensus process.	0.355	1.8
Recommendations	0.170	31. Make the recommendations clearly identifiable (e.g., in a table, or using enlarged or bold fonts).	0.167	4.1
		32. Indicate the strength of all recommendations.	0.240	6.3
		33. Provide the explanations for all recommendations.	0.367	3.9
		34. Indicate the considerations (e.g., adverse effects) in clinical practice when implementing the recommendations.	0.231	2.8
Accessibility	0.073	35. Make the guideline accessible through multiple platforms (e. g., guideline libraries, conference presentations, and websites).	0.349	2.5
		36. Provide tailored editions of the guidelines for different groups of target users (e.g., patients, public, primary care physicians).	0.186	1.4
		37. Present the guideline or recommendations visually, such as with figures or videos.	0.152	1.1
		38. Make the full guideline downloadable free of charge.	0.314	2.3
Other	0.012	39. Provide a flowchart of clinical pathways reflecting the recommendations.	1.000	1.2

From: Yang N, Liu H, Zhao W et al (2023) Development of the Scientific, Transparent and Applicable Rankings (STAR) tool for clinical practice guidelines. Chin Med J (Engl) 136(12):1430-1438

Supplementary Table S3 STAR scoring of each checklist

Item	ESUR_2018	RANZCR_2018	JRS-JCS-JSN_2018	UCSF-USC_2020	ACR-NKF_2021	CSR_2021	CSCP-CPA-CSN_2022	SIRM-SIN-AIOM_2022	CAR_2022	ACR_2023
1	0	0	0	0	0	0	0	0	0	0
2	0	0	0	0	0	0	0	0	0	0
3	0	0	0	0	0	0	0	0	0	0
4	0	0	0	0	0	0	0	0	0	0
5	1	1	1	0	0.5	0	0	1	1	0
6	1	1	0	0	0	0	0	1	1	0
7	1	1	0	0	0	0	0	0	1	0
8	1	1	1	1	1	1	1	1	1	1
9	0	1	0.5	0	0	1	1	0	0	0
10	0	1	0	0	1	0	0	0	0	0
11	0	1	1	0	0.5	0	1	1	0.5	0
12	0.5	1	0	0	0	0	0	0	0	0
13	1	1	1	1	1	1	1	1	1	0
14	0	0	0	1	0	1	1	1	1	0
15	1	1	1	1	1	1	1	1	1	1
16	1	1	1	0.5	1	1	1	0.5	1	1
17	0.5	1	0.5	0	0.5	0.5	0.5	0	0	1
18	1	1	1	0.5	0.5	1	0	0.5	0	1
19	1	1	1	1	1	1	1	1	1	1
20	1	1	0	0	0	0	0	0	0	0
21	1	1	0	0	0	0	0	0	0	0
22	1	1	1	0	0	0	0	0	1	1
23	0	0	0	0	0	0	0	0	0	0
24	1	0	1	0	0	0	1	0	0	1
25	0.5	0	0.5	0	0	0	0	0	0.5	0.5
26	1	0	0	0	0	0	0	0	0	0
27	1	1	1	1	1	1	1	1	1	1
28	0	1	1	0	0	0	0	0	0	0
29	0.5	0	0	0	0	0	0	0	0.5	0
30	0	0	0	0	0	0	0	0	0	0
31	1	1	1	0.5	1	0.5	1	1	1	1
32	1	0	1	0	0	0	1	0	0	1
33	1	1	1	0.5	1	0.5	1	1	1	1
34	1	1	1	0.5	1	0.5	1	1	1	1
35	0.5	0	0.5	0	0.5	0	0	0.5	0.5	0
36	0	0	0	0	0	0	0	0	0	0
37	0	0	0	0	0	0	0	0	0	0
38	1	1	1	0	1	0	1	1	1	1
39	0	0	0	0	0	1	0	0	1	0

Supplementary Table S4 List of discussed recommendations

Guideline	Recommendation
**Screening Patients with Regard to Their Need for Renal Function Testing**	
RANZCR_2018	❿ Prior to intravascular administration of iodinated contrast media patients should be asked the following. If present, an eGFR should be obtained prior to iodinated contrast media administration in non-emergency patients: (a) known kidney disease (including kidney transplant); (b) presence of diabetes; (c) whether they are currently taking a drug containing metformin.<br>❿ Non-anuric patients currently on short- or long-term dialysis may require consultation with a renal physician prior to iodinated contrast media administration.<br>❿ Age should not be considered as an independent risk factor that should mandate testing as eGFR declines with age even in healthy individuals, due to the way it is calculated.<br>❿ The time elapsed between renal function testing and contrast media administration should be governed by clinical judgment based upon the likelihood that renal function has deteriorated to a clinically significant degree since the renal function was assessed.
ACR_2023	❿ Screening based on eGFR should be used to identify patients at potential risk of PC-AKI: (a) Personal history of renal disease, including: known chronic kidney disease (CKD), remote history of AKI, dialysis, kidney surgery, kidney ablation, albuminuria; (b) history of diabetes mellitus (optional); (c) metformin or metformin-containing drug combinations.
UCSF-USC_2020	❿ Patients should be assessed using a standardized “Patient Screening Form” to indicate relevant medical history and history of previous reactions to iodinated contrast: (a) Screening form questions: Have you ever had a previous reaction to intravenous contrast? Have you ever had a life-threatening allergic reaction (both medications and food)? (b) For women of child-bearing age: Is there a possibility that you may be pregnant? Are you breastfeeding? If answered yes to any of the questions above, consultation with supervising radiologist recommended.<br>❿ Screening questions to determine need for further renal studies: What is your current age? Do you have diabetes? Do you have high blood pressure requiring medication? Do you have ANY problems with your kidneys (i.e., transplant, single kidney, renal cancer, dialysis, kidney surgery)? If < 60 years old and answered NO to questions 2–4, IV contrast can be administered without laboratory workup for renal function. If > 60 years old and/or answered YES to any of the questions, assess renal function with laboratory workup prior to administering contrast.<br>❿ Routine laboratory assessment of renal function for all patients prior to administration of contrast is not recommended.
ACR-NKF_2021	❿ Screening based on eGFR should be used to identify patients at potential risk of PC-AKI: (a) Personal history of renal disease, including: known chronic kidney disease (CKD), remote history of AKI, dialysis, kidney surgery, kidney ablation, albuminuria; (b) history of diabetes mellitus (optional); (c) metformin or metformin-containing drug combinations.
CAR_2022	❿ For stable outpatients without a current (3- 6 months depending on institutional preference) eGFR on file, and those without a provided history of CKD on the requisition, We recommend a simple screening questionnaire to detect those who may have AKI or severe CKD: “Do you have kidney problems or a kidney transplant?” “Have you seen, or are you waiting to see a kidney specialist or urologist (kidney surgeon)?” (a) When the patient or substitute decision maker (SDM) answers YES to the either question they should have blood work drawn for a current eGFR before protocoling the study with ICM. (b) If the patient (or SDM) answers NO then an indicated ICM examination can proceed without a current eGFR value.
CSCP-CPA-CSN_2022	❿ It is still recommended that patients over 60 years old undergo renal function assessment before using iodine contrast agents.<br>❿ It is not recommended to use gender as an influencing factor in evaluating CI-AKI.<br>❿ Hyperuricemia may be associated with an increased risk of CI - AKI<br>❿ Diabetes is a risk factor for CI - AKI, but it is not recommended to use it as an independent risk factor for assessing the risk of CI - AKI.
EUSR_2018	❿ In hospitals which use sCr measurements for all patients before intravascular CM administration there is no benefit in using questionnaires for PC-AKI risk stratification.

	⓾ In hospitals which use sCr measurements selectively, Choyke questionnaires may be used to identify patients with eGFR < 45 ml/min/1.73 m2 before intra-arterial CM administration with first pass renal exposure.
JRS-JCS-JSN_2018	⓾ CKD (eGFR < 60 mL/min/1.73 m) is a risk factor for the development of CIN. However, the risk depends on the administration route of the contrast media and the pathophysiological condition of the patient. Aging is a risk factor for the development of CIN. Although diabetes mellitus associated with CKD (eGFR < 60 mL/min/1.73 m2) is a risk factor for the development of CIN, it is unclear whether diabetes mellitus without CKD is a risk factor. There is no evidence that RAS inhibitors increase the risk of developing CIN. We recommend against the use of NSAIDs owing to an increased risk of developing CIN.
**Clinical Estimation of Renal Function**	
RANZCR_2018	⓾ eGFR using the CKD-EPI formula using serum creatinine, patient age, gender and race, should be used in preference to serum creatinine to identify patients with severely impaired renal function.<br>⓾ eGFR should not be relied upon as an accurate indicator of renal function in patients who are known to have acute kidney injury for any reason.
ACR_2023	⓾ eGFR is gaining attention as a potentially better marker of PC-AKI risk
UCSF-USC_2020	⓾ In patients who have a known history of renal impairment, a recent creatinine or eGFR within the past 6 weeks for outpatients or last 7 days for inpatients is considered appropriate for evaluation of renal function at the authors' institution.
ACR-NKF_2021	⓾ Screening based on eGFR should be used to identify patients at potential risk of CI-AKI.
CAR_2022	⓾ We recommend a current eGFR (within 7 days for inpatients, or upon presentation for ER patients), however this should not delay emergent imaging examination. (a) Emergent presentation. When the patient is in an emergent presentation (such as suspected acute stroke, pulmonary embolism, acute aortic syndrome, bowel ischemia or perforation, and other conditions) an indicated contrast enhanced imaging study should proceed without delay. Do not delay for eGFR and do not withhold contrast that is necessary for an accurate diagnosis of the emergent pathology. (b) Use of intravenous or intra-arterial contrast in the setting of pre-existing AKI should consider the trade-off of overall risk of worsening AKI with contrast against the benefit of improved diagnostic capability and therapeutic intervention.
CSCP-CPA-CSN_2022	⓾ In non-emergency situations, it is recommended to conduct renal function testing within 7 days before performing iodine contrast agent examination; In emergency situations, if iodine contrast agent testing can be postponed, it is recommended to conduct renal function testing first; It is still recommended to conduct renal function testing first in cases where iodine contrast agent testing may cause harm to patients if not performed immediately. However, iodine contrast agent testing can be performed urgently in the absence of renal function test results.
CSR_2021	⓾ It is recommended to measure sCr levels before CT enhanced examination in CKD patients to evaluate renal function.
ESUR_2018	⓾ GFR is estimated using sCr as an endogenous filtration marker<br>⓾ The CMSC considers eGFR measurements before intra- vascular CM exposure valid for a maximum of: 7 days* if the patient (a) has an acute disease, an acute deterioration of a known chronic disease or any other adverse event that could have negatively influenced renal function (eGFR), or (b) is a hospital inpatient. 3 months (a) if the patient has a chronic disease with stable renal function (eGFR), and (b) in all other patients
SIRM-SIN-AIOM_2022	⓾ Serum creatinine alone is not a good index of a patient's renal function because its value increases significantly only when the GFR is reduced to 50%<br>⓾ The GFR value obtained from serum creatinine with the MDRD formula or the CKD—EPI formula is the marker of renal function to use in screening patients for renal risk. The ESUR GLs recommended the use of the CKD—EPI formula. The GFR measurement obtained from the two formulas is normalized to 1.73 m2 of body surface area and does not consider the patient's body weight<br>⓾ Renal filtrate can be measured with the Cockroft–Gault formula in patients with significantly reduced body weight<br>⓾ We recommend assessing baseline eGFR on creatinine value per- formed within 7 days in patients

	with unstable or hospitalized renal function, whereas a 3-month interval is considered correct in other patients<br>➓ Chronic renal failure is considered the major risk factor in the onco- logical patient, but only for an eGFR < 30 ml/minute/1.73 m2 as measured by the Cockroft–Gault formula
**Contraindications for Risk Stratification of PC-AKI**	
RANZCR_2018	➓ Intravascular iodinated contrast media should be given to any patient regardless of renal function status if the perceived diagnostic benefit to the patient, in the opinion of the radiologist and the referrer, justifies this administration.<br>➓ Emergency imaging procedures requiring contrast media administration e.g., acute stroke, acute bleeding, trauma etc. should not be delayed in order to obtain renal function testing results prior to the procedure.<br>➓ The risk of intravenous contrast media related acute kidney injury (CI-AKI) is likely to be non-existent for patients with eGFR greater than 45 mL/min/1.73m^2. No special precautions are recommended in this group prior to or following intravenous administration of iodinated contrast media.<br>➓ The risk is of intravenous CI-AKI is also very likely to be low or non-existent for patients with eGFR 30 - 45 mL/min/1.73m2. Universal use of periprocedural hydration in this group to prevent the theoretical risk of CI-AKI cannot be recommended but patients with impaired function in this range that is acutely deteriorating rather than stable may benefit from this intervention.<br>➓ In patients with severe renal function impairment (eGFR less than 30 ml/min/1.73m2) or actively deteriorating renal function (acute kidney injury) careful weighing of the risk versus the benefit of iodinated contrast media administration needs to be undertaken. Consideration should be given to periprocedural renal protection using intravenous hydration with 0.9% saline (see relevant section). However, severe renal function impairment should not be regarded as an absolute contraindication to medically indicated iodinated contrast media administration.
ACR_2023	➓ If a threshold for CI-AKI risk is used at all, 30 mL / min/1.73m2 seems to be the one with the greatest level of evidence. Any threshold put into practice must be weighed on an individual patient level with the benefits of administering contrast material.
UCSF-USC_2020	➓ The evidence demonstrates that patients with an eGFR > 30 do not have an increased risk of developing post-contrast AKI. It remains unclear whether patients with an eGFR < 30 are at an increased risk of developing post-contrast AKI.
ACR-NKF_2021	➓ Patients with AKI or eGFR less than 30 mL/min/1.73 m2 (including non-anuric patients undergoing maintenance dialysis) should prompt consideration by the referring professional and radiologist to discuss the risks and benefits of contrast media ad- ministration.
CAR_2022	➓ For non-emergent presentation of stable outpatients, inpatients, and emergency patients when eGFR is available. (a) If eGFR >30 mL/min/1.73 m2 and no signs and symptoms of AKI, then proceed with an indicated contrast imaging study. If eGFR ≤30 mL/min/1.73 m2, or suspected AKI We recommend an individual patient decision involving the caring team, or patient/patient decision-maker to explain and balance the risks of CA-AKI against the risks and uncertainties of delayed or suboptimal imaging. (b) Imaging with ICM can be performed in patients on peritoneal or hemodialysis regardless of residual urine output and no change in dialysis schedule is required.
CSCP-CPA-CSN_2022	➓ Patients with AKI or eGFR less than 30 mL/min/1.73 m2 is risk factor for CI-AKI.
CSR_2021	➓ The recommended eGFR risk threshold for patients undergoing enhanced CT examination is 30 ml (/min · 1.73 m2). Based on existing evidence, it is safe to directly perform enhanced examination for patients with eGFR ≥ 30ml (/min · 1.73m2). For patients with eGFR of 30-44ml (/min · 1.73m2) and high-risk factors and<30ml (/min · 1.73m2), the use of iodine contrast agents can be considered comprehensively, and the relevant information can be explained to the patient before examination before appropriate use.
ESUR_2018	➓ The risk of PC-AKI in patients with eGFR ≥ 30 ml/min/1.73m2 after intravenous and intra-arterial CM administration with second-pass renal exposure is very low, but there is conflicting evidence on the risk for intra-arterial CM administration with first-pass renal exposure<br>➓ Preventive measures are recommended for patients with eGFR < 30 ml/min/1.73m2 before

	intravenous and intra-arterial CM administration with second-pass renal exposure<br>❿ Preventive measures are recommended for patients with eGFR < 45 ml/min/1.73m2 if they are in ICU or if they will receive intra-arterial CM administration with first-pass renal exposure<br>❿ Recommendations for prevention of PC-AKI in adults may also be used in children and adolescents<br>❿ The principal risk factor for PC-AKI is impaired renal function. Most other published patient-related risk factors are risk factors for the presence of chronic kidney disease or AKI, and are not specific for PC-AKI
JRS-JCS-JSN_2018	❿ It is unlikely that the risk of developing CIN increases in CKD patients (eGFR ⩾ 30 mL/min/1.73 m2) after contrast-enhanced CT. However, even if the eGFR is ⩾ 30 mL/min/1.73 m2, it is important to fully evaluate the risk factors for CIN (see Chapter 3). On the other hand, when contrast-enhanced CT is performed in CKD patients with an eGFR < 30 mL/min/1.73 m2, it is recommended that the risk of CIN onset be explained, and appropriate preventive measures be taken as necessary.<br>❿ In intensive care and severe emergency outpatient patients, there is little evidence that contrast-enhanced CT is a risk factor for developing CIN. However, in these patients, the risk of developing AKI is high irrespective of the administration of contrast media. Therefore, when contrast-enhanced CT is performed, it is recommended to sufficiently explain AKI and CIN and to take appropriate preventive measures.
**Peri-administration Strategies to Mitigate the Risk of PC-AKI in Higher Risk Individuals**	
RANZCR_2018	❿ For patients who are at higher risk of PC-AKI, pre and post procedural 0.9% IV saline is recommended as the first line preventive strategy to mitigate the risk of CI-AKI.<br>❿ The evidence in support of the additional benefit of N–acetyl cysteine and/or sodium bicarbonate alone or in combination with intravenous 0.9% saline is mixed and currently these additional measures are not recommended due to additional expense and complexity without clear evidence of incremental risk reduction.
ACR_2023	❿ Concern for the development of PC-AKI is a relative but not absolute contraindication to the administration of intravascular iodinated contrast medium in at-risk patients that have AKI or an eGFR less than 30 mL/min/1.73 m2 and are not undergoing maintenance dialysis.<br>❿ It is not recommended to reduce doses to attempt to mitigate the risk of CI-AKI as this may result in suboptimal or nondiagnostic images. Instead, standard contrast dosing is recommended if the benefits have been deemed to outweigh the risks for intravenous iodinated contrast media administration in high-risk patients for PC-AKI.<br>❿ We do not believe that there is sufficient evidence to specifically endorse the decision to withhold a repeat contrast medium injection until more than 24 hours have passed since the prior injection, nor to recommend a specific threshold of contrast medium volume beyond which additional contrast media should not be given within a 24-hour period. The decision to administer closely spaced contrast-enhanced studies is clinical and subjective, with high- risk patients (e.g., Stage IV and Stage V chronic kidney disease, AKI) treated with greater caution than the general population.
UCSF-USC_2020	❿ In patients with eGFR ⩾ 30 mL/min/1.73 m2. Low risk: Limited evidence suggesting intravenous iodinated contrast material is an independent risk factor for AKI in patients with eGFR ⩾ 30 mL/min/1.73 m2. No hydration required.<br>❿ In patients with eGFR < 30 mL/min/1.73 m2. Higher risk. This population of patients has the greatest risk for post-contrast acute kidney injury after administration of intravenous iodinated contrast. Contrast is not recommended unless the patient is on dialysis and anuric, or if contrast is considered diagnostically essential. Weigh the benefits of contrast versus potential harms of kidney injury. Pre-procedural prophylaxis with intravenous volume expansion therapy is recommended. The optimal IV volume expansion protocol is unknown and ideally should be tailored to the patient's volume status and medical conditions. Suggested protocols: (a) Inpatients—0.9% normal saline at 100 mL/h IV beginning 6–12 h prior to contrast administration and continuing 4–12 h afterwards. (b) Outpatients—0.9% normal saline 500 mL IV bolus prior to contrast administration. Consider post-exposure oral hydration (1 cup of water per hour for 8 h) unless medically contraindicated.

ACR-NKF_2021	❿ Patients with CKD stages 4 or 5 have a relative rather than absolute contraindication to iodinated contrast media. If contrast media administration is required for a life-threatening diagnosis, then it should not be withheld based on kidney function. If intravenous iodinated contrast media administration is clinically indicated, then its use should be informed by consideration of the potential risks and benefits as well as alternative imaging strategies. If the decision is made to administer iodinated contrast media in this setting, then volume expansion with normal saline is indicated if there are no contraindications<br>❿ Prophylaxis is indicated for patients who have AKI or an eGFR less than 30 mL/min/1.73 m2 and are not undergoing maintenance dialysis. However, the evidence sup- porting this statement is based on data for the general prevention of CA-AKI rather than CI-AKI specifically. The risks of prophylaxis (e. g., heart failure, other hypervolemic conditions) should be considered before initiation. Prophylaxis is not indicated for the general population of patients with stable eGFR greater than or equal to 30 mL/min/1.73m2, for patients undergoing chronic dialysis, or for patients at risk for heart failure. This eGFR threshold should not be adjusted solely based on concomitant diabetes mellitus.
CAR_2022	❿ We do not recommend preferential use of iso-osmolar ICM for reducing risk of CA-AKI; those decisions should be made based on other factors (e.g., cost and availability).<br>❿ We discourage reduced dosing of IV contrast administration for CT examinations since that lowers parenchymal enhancement and recommend using the appropriate IV dose for high quality CT imaging in all patients.<br>❿ We recommend a pragmatic approach to ICM dosing for IA interventions using the necessary dose to achieve the diagnostic and therapeutic results but judiciously reducing dose when adjunctive imaging and doses are low yield or can be delayed.<br>❿ We do not recommend restricting repeat contrast doses in lower risk patients (eGFR >30, no AKI, IV route) or withholding repeat doses for emergency or inpatients who have life-threatening, or acute presentation of illness. We do recommend avoiding repeated contrast exposures within 48 hours for elective procedures if the patients at higher risk of CA-AKI (eGFR ≤30, AKI, intraarterial ICM administration). However, in the face of life- threatening illness, repeat dosing of ICM may be necessary and justified to establish a confident diagnosis and treatment plan.<br>❿ We do not recommend oral or intravenous hydration for patients with eGFR >30 mL/min/1.73m2, receiving intravenous or intra-arterial ICM.<br>❿ For patients with eGFR ≤30 mL/min/1.73m2, receiving intravenous ICM, there is a lack of evidence on benefit of volume expansion. Hence the working group makes no recommendation in this regard; institutions may choose practices best suited to their local environments.<br>❿ For patients with eGFR ≤30 mL/min/1.73m2, receiving intra-arterial ICM, some members of the working group endorsed a strategy of hydration and volume expansion using either intravenous hydration (with .9% saline or 1.26% sodium bicarbonate) or oral salt and water. Since there is insufficient evidence in this patient group the working group felt the use of hydration or not, and the route of hydration was best left to judgement of the practitioner.<br>❿ We do not recommend preferential use of iso- osmolar ICM for reducing risk of CA-AKI. We recommend decisions about low-osmolar or iso- osmolar ICM be made based on other factors (e.g., cost and availability).<br>❿ We do not recommend any form of post ICM administration renal replacement therapy, either dialysis or continuous renal replacement therapy for reduction of the risk of CA-AKI.<br>❿ We do not recommend N-acetylcysteine use for the prophylaxis of CA-AKI.<br>❿ We do not recommend initiating statins specifically for prevention of CA-AKI.<br>❿ We do not recommend use of other pharmacological agents which have been described in the literature, including theophylline, prostaglandin E1, nicorandil, ascorbic acid, allopurinol, alpha-tocopherol, fenoldopam, natriuretic peptides, and trimetazidine.<br>❿ We recommend a follow-up serum creatinine measurement 48 to 72 hours after intra-arterial ICM injection in all patients with eGFR ≤30 mL/min/ 1.73m2. For the remainder of patients, the risk of AKI is extremely low, and routine testing is not warranted. However, any at-risk patient should be instructed to seek medical attention and kidney function testing if they develop

	increased shortness- of-breath, peripheral edema, or note a marked decline in urine output in the days following the imaging test.
CSCP-CPA-CSN_2022	❿ The risk of CI - AKI in hypo-hypertonic and isotonic iodine contrast agents is lower than that in hypertonic iodine contrast agents. It is recommended to use non-ionic hypo-hypertonic or isotonic iodine contrast agents, and the use of ionic hypertonic iodine contrast agents is not recommended.<br>❿ There is currently no clear conclusion on whether the risk of CI - AKI varies among different varieties of isotonic iodine contrast agents or sub hypertonic iodine contrast agents.<br>❿ For patients at high risk of CI - AKI, renal function should be rechecked within 48 hours after receiving iodine contrast agent examination. If CI - AKI is diagnosed within 48 hours, early intervention measures should be taken and the patient's renal function should be monitored for at least 30 days.<br>❿ The dosage of iodine contrast agent is a risk factor for CI - AKI. It is recommended to minimize the dosage of iodine contrast agent while meeting clinical diagnosis and treatment needs.<br>❿ 17. The repeated use of iodine contrast agents in PCI patients for a short period of time (48-72 hours) is a risk factor for CI AKI. When there are no other risk factors present, as long as the gI/eGFR does not exceed 1.0, iodine contrast agents can be repeatedly injected; When there are other risk factors, it is best to repeatedly inject iodine contrast agent at a minimum interval of 48 hours and test renal function before this.<br>❿ If the patient does not have contraindications for volume expansion, it is recommended to use hydration to prevent CI - AKI.<br>❿ Oral hydration is not recommended as the first or only preventive strategy<br>❿ Both physiological saline and sodium bicarbonate solution can be used as hydration crystal solutions, and suitable hydration crystals can be selected according to clinical needs<br>❿ The advantages and disadvantages of different hydration schemes are still unclear, and further research is needed on the optimal individualized hydration scheme (such as the speed, volume, and time of intravenous infusion).<br>❿ The effectiveness of NAc in preventing CI - AKI is still unclear.<br>❿ Short term high-dose statins may have potential preventive effects on CI AKI, but statins are not recommended as a routine preventive strategy for CI AKI.<br>❿ Vitamin C may have potential preventive effects on CI - AKI, but clinical research is still needed to confirm.<br>❿ The preventive effect of RAAS inhibitors on CI - AKI is unclear, and it is not recommended to use RAAS inhibitors as a routine preventive strategy for CI - AKI<br>❿ It is not recommended to use conventional dialysis treatment for CI - AKI. Dialysis treatment can only be considered in cases where the condition is seriously life-threatening and there are indications for dialysis.
CSR_2021	❿ Ionic high permeability contrast agents increase the risk of P C-A K I, and it is recommended to use IOCM or LOCM. It is recommended to choose iodine contrast agents supported by evidence-based evidence. While maintaining image quality, the amount of iodine contrast agent used can be moderately reduced to avoid delaying diagnosis due to a significant decrease in image quality caused by the use of iodine contrast agent. It is not recommended to repeat CT enhanced examination within 3 days.<br>❿ Patients with eGFR<30ml (/min · 1.73m2) who have not undergone maintenance dialysis should carefully evaluate factors such as heart function before undergoing intravenous hydration prevention in clinical practice. For patients with stable eGFR and ≥ 30 ml (/min · 1.73 m2), generally no preventive measures need to be taken. If the patient has multiple risk factors and the eGFR range is 30-44 ml (/min · 1.73 m2), appropriate preventive measures can be taken based on the physician's judgment.
EUSR_2018	❿ There is no difference in PC-AKI risk between IOCM and LOCM. The use of ionic, high-osmolar CM and repeated CM injections in a short period (48–72 h) should be avoided<br>❿ When CM are injected intravenously, there is insufficient evidence that CM dose is a risk factor.

	When CM are injected intra-arterially, the ratio of CM dose (in gram Iodine) / absolute eGFR (in ml/min) should be kept below 1.1 or the ratio of CM volume (in ml) / eGFR (in ml/min/1.73m2) should be kept below 3.0 when using a CM concentration of 350 mg/ml<br>❿ Preventive hydration should be used to reduce the incidence of PC-AKI in at-risk patients. Intravenous saline and bicarbonate protocols have similar efficacy for hydration. For intravenous and intra-arterial CM administration with second pass renal exposure hydrate the patient with either (a) 3 ml/kg/h bicarbonate 1.4% (or 154 mmol/l solution) for 1 h before CM or (b) 1 ml/kg/h saline 0.9% for 3–4 h before and 4–6 h after CM. For intra-arterial CM administration with first pass renal exposure hydrate the patient with either (a) 3 ml/kg/h bicarbonate 1.4% (or 154 mmol/l solution) for 1 h before CM followed by 1 ml/kg/h bicarbonate 1.4% (or 154 mmol/l) for 4–6 h after CM or (b) 1 ml/kg/h saline 0.9% for 3–4 h before and 4–6 h after CM. Oral hydration as the sole means of prevention is not recommended. In patients with severe heart failure (NYHA grade 3–4) or patients with end-stage renal failure (CKD grade V) preventive IV hydration should be individualized by the clinician responsible for patient care.<br>❿ N-Acetylcysteine has not been conclusively shown to reduce the risk of PC-AKI in patients with eGFR < 45 ml/min/1.73 m2 receiving intravenous or intra-arterial CM, and its use is NOT recommended. Giving short-term, high-dose statins to patients not already taking statins has not been shown to reduce the risk of PC-AKI in patients with eGFR < 45 ml/min/1.73 m2 receiving intravenous or intra-arterial CM, and its use is NOT recommended. ACE inhibitors or angiotensin receptor blockers have not been shown conclusively to reduce the risk of PC-AKI in patients receiving intravenous or intra-arterial CM, and their use is NOT recommended. Vitamin C has not been shown conclusively to reduce the risk of PC-AKI in patients receiving intravenous or intra-arterial CM, and its use is NOT recommended.<br>❿ Renal replacement therapy has not been shown conclusively to reduce the risk of PC-AKI in patients receiving intravenous or intra-arterial CM, and its use is NOT recommended.<br>❿ It is not necessary to adapt the timing of intravascular CM administration in relation to the dialysis schedule in patients undergoing chronic dialysis or haemofiltration, but it may be done to minimise volume overload.
JRS-JCS-JSN_2018	❿ The risk of developing CIN does not differ between iso-and low-osmolar contrast media. Although there has been no definitive conclusion as to whether the risk of developing CIN differs among the different types of low-osmolar contrast media, there has been no significant difference in the incidence of CIN among them.<br>❿ There is a possibility that a reduction in the contrast media volume in contrast-enhanced CT may reduce the risk of developing CIN. For patients with a high CIN risk (CQ 6-1, 2) in particular, it is recommended to utilize the minimum amount of contrast media necessary for diagnostic efficacy. When decreasing the contrast media usage, it is recommended to combine low tube voltage imaging and iterative reconstruction in facilities where it is possible.<br>❿ Since repeated contrast-enhanced CT at short intervals may increase the risk of developing CIN, we do not recommend performing a repeat contrast-enhanced CT within 24–48 h of the first.<br>❿ We recommend using physiological saline intravenously before and after contrast-enhanced examination in CKD patients, as they are at high risk of developing CIN.<br>❿ We recommend using isotonic solutions to prevent CIN because isotonic 0.9% sodium chloride (physiological saline) is superior to hypotonic 0.45% sodium chloride in preventing CIN.<br>❿ There is no sufficient evidence that oral water intake is as effective as intravenous hydration therapy in preventing the development of CIN. We recommend that patients receive hydration therapy or other established preventive measures rather than relying on oral water intake to prevent CIN.<br>❿ Sodium bicarbonate-based hydration may decrease the risk of developing CIN. When infusion time is limited, administration of sodium bicarbonate-based hydration is recommended.<br>❿ There is no conclusive evidence that short-term intravenous sodium bicarbonate hydration is as effective as standard intravenous hydration for preventing CIN. Excluding emergency cases with limited infusion time, it is recommended to administer infusion for an extended period of time.<br>❿ We do not recommend the use of NAC for the prevention of CIN onset. We do not recommend

	the use of hANP for prevention for CIN onset. We do not recommend the use of ascorbic acid for the prevention of CIN. We do not recommend the use of statins for the prevention of CIN onset.<br>➓ Blood purification therapy after administering contrast media does not decrease the risk of developing CIN and is not recommended. In particular, hemodialysis therapy is not recommended.
SIRM-SIN-AIOM_2022	➓ Hydration/volume expansion represents the gold standard of PC-AKI preventive therapy, keeping in mind that any intervention must be proportionate to the patient's overall risk;<br>➓ Intravenous hydration is recommended as a preventive measure for patients with moderate risk of PC-AKI (intravenous or intra-arterial administration with second renal passage with GFR < 30) using either Na bicarbonate (NaBic) 1.4% 3 ml/Kg/hr for 1 h prior to administration, or saline 1 ml/Kg/hr for 3–4 h before and 4–6 h after administration.<br>➓ Two meta-analyses by Meier show that NaBic is more useful than saline when there is no time to perform prolonged hydration and thus may be more appropriate in emergency procedures<br>➓ Hydration is the only recommended measure to prevent PC-AKI from contrast agent infusion even in the high-risk oncological patient;<br>➓ Oncological patients treated with potentially nephrotoxic chemo- therapy (especially if containing cisplatin) who receive iodine- based contrast agent are at high risk of developing PC-AKI. It is recommended that 5 to 7 days elapse between the administration of cisplatin and the contrast agent. There are no data in the literature regarding other chemotherapeutics, which are therefore not recommended to be discontinued before a CT with contrast agent;<br>➓ In patients treated with molecularly targeted drugs and immuno- therapy, renal damage due to these drugs has a significantly lower incidence than with chemotherapy; it is not recommended to discontinue treatment at CT scans with contrast agent<br>➓ In patients treated with bisphosphonates, it is recommended that an adequate interval be maintained between their administration and that of the contrast agent. A 14-day interval between zoledronic acid and iodine-based agent administration is recommended

From:

[1] European Society of Urogenital Radiology (2018) ESUR Guidelines on contrast agents. Available via https://www.esur.org/wp-content/uploads/2022/03/ESUR-Guidelines-10_0-Final-Version.pdf Accessed 15 July 2023.

[2] The Royal Australian and New Zealand College of Radiologists (2018) Iodinated contrast media guideline, version 2.3. Available via https://www.ranzcr.com/search/ranzcr-iodinated-contrast-guidelines Accessed 15 July 2023.

[3] Isaka Y, Hayashi H, Aonuma K et al; Japanese Society of Nephrology, Japan Radiological Society, Japanese Circulation Society Joint Working Group (2020) Guideline on the use of iodinated contrast media in patients with kidney disease 2018. Jpn J Radiol 38(1):3-46

[4] Huynh K, Baghdanian AH, Baghdanian AA, Sun DS, Kolli KP, Zagoria RJ (2020) Updated guidelines for intravenous contrast use for CT and MRI. Emerg Radiol 27(2):115-126

[5] Davenport MS, Perazella MA, Yee J et al (2020) Use of intravenous iodinated contrast media in patients with kidney disease: consensus statements from the American College of Radiology and the National Kidney Foundation. Radiology 294(3):660-668

[6] Quality Control and Safety Management Committee of Chinese Society of Radiology Chinese Medical Association (2021) Expert consensus of iodinated contrast agent use in patients with renal diseases. Chin J Radiol 55(6):580-590 [Article in Chinese]

[7] Chinese Society of Clinical Pharmacy, Hospital Pharmacy Professional Committee of Chinese Pharmaceutical Association, Chinese Society of Nephrology (2022) Expert consensus on prevention and treatment of iodine contrast media-induced acute kidney injury. Chin J Nephrol 38(3):265-288 [Article in Chinese]

[8] Orlacchio A, Guastoni C, Beretta GD et al (2022) SIRM-SIN-AIOM: appropriateness criteria for evaluation and prevention of renal damage in the patient undergoing contrast medium examinations-consensus statements from Italian College of Radiology (SIRM), Italian College of Nephrology (SIN) and Italian Association of Medical Oncology (AIOM). Radiol Med 127(5):534-542

[9] Macdonald DB, Hurrell C, Costa AF et al (2022) Canadian Association of Radiologists guidance on contrast associated acute kidney injury. Can Assoc Radiol J 73(3):499-514

[10] American College of Radiology (2023) ACR manual on contrast media. Available via https://www.acr.org/Clinical-Resources/Contrast-Manual Accessed 15 July 2023

Supplementary Table S5 List of answers for interested questions

Guideline	Recommendation
**Who should undergo renal function test before contrast media administration?**	
ESUR_2018	Risk factor: impaired renal function.<br>(1) In hospitals which use sCr measurements for all patients before intravascular CM administration there is no benefit in using questionnaires for PC-AKI risk stratification.<br>(2) In hospitals which use sCr measurements selectively, Choyke questionnaires (a history of renal disease or renal surgery [eGFR < 60 ml/min/1.73 m2], heart failure, diabetes, proteinuria, hypertension and gout) may be used to identify patients with eGFR < 45 ml/min/1.73 m2 before intra-arterial CM administration with first pass renal exposure.
RANZCR_2018	Should be obtained in patients with (a) known kidney disease; (b) diabetes; (c) metformin.<br>May required: Non-auric patients on dialysis.<br>Should not be considered: Aging
JRS-JCS-JSN_2018	No recommendation.<br>Risk factor: < 60, aging<br>Unclear: diabetes, RASI, NSIAD
UCSF-USC_2020	Need for > 60 years old, diabetes, high blood pressure with medication, kidney problems.
ACR-NKF_2021	Should be obtained in patients with (a) known kidney disease; (b) metformin.<br>Optional: diabetes
CSR_2021	CKD patients.
CSCP-CPA-CSN_2022	Recommended: > 60 years old<br>Not recommended: Gender, diabetes,<br>May be associated: hyperuricemia
SIRM-SIN-AIOM_2022	No recommendation.<br>Risk factor: renal insufficiency, diabetes, advanced age, heart failure, hypovolemia, myocardial infarction, anemia, peripheral vasculopathy, and the use of nephrotoxic drugs (some antibiotics, some anticancer drugs, NSAIDs, cyclosporine); presence of pre- and intra-procedure hypotension, the use of a cardiac counter-pulsator, and multiple, close (< 72 h) administrations; hyperglycemia, metabolic syndrome, hyperuricemia, hyper-homocysteinemia, atrial fibrillation.<br>No evidence for ACE inhibitor drugs or angiotensin receptor blockers.
CAR_2022	Should have: kidney problem/ waiting for transplant/ seeing a kidney specialist
ACR_2023	Should be obtained in patients with (a) known kidney disease; (b) metformin.<br>Optional: diabetes
**How long is the time interval between renal function test and administration acceptable?**	
ESUR_2018	Within 7 days for patient has an acute disease, an acute deterioration of a known chronic disease or any other adverse event that could have negatively influenced renal function, or for inpatient.<br>Within 3 months for patient has a chronic disease with stable renal function (eGFR), and in all other patients.
RANZCR_2018	Depends on clinical judgment based upon the likelihood that renal function has deteriorated to a clinically significant degree since the renal function was assessed.<br>The maximum safe interval between an eGFR result and contrast media administration is unknown, with various guidelines recommending intervals of 7 days (ACR), 30 days (ESUR), 3 months (RCR and RANZCR 2009) and 6 months (CAR) as "consensus" rather than evidence-based recommendations.
JRS-JCS-JSN_2018<br>(Follows ESUR_2018)	With regard to the timing of eGFR measurement, the ESUR has recommended measurement within 7 days in patients with acute disease, inpatients, and patients at high risk of CIN and within 3 months in patients with stable renal function.<br>When contrast-enhanced CT is performed emergency, it is recommended to sufficiently explain AKI and CIN and to take appropriate preventive measures.
UCSF-USC_2020	Within past 6 weeks for outpatients, or past 7 days for inpatients with renal impairment.

ACR-NKF_2021	No recommendation.
CSR_2021<br>(Follows ESUR_2018)	Within 7 days for patient has an acute disease, an acute deterioration of a known chronic disease or any other adverse event that could have negatively influenced renal function, or for inpatient.<br>Within 3 months for patient has a chronic disease with stable renal function (eGFR), and (b) in all other patients.
CSCP-CPA-CSN_2022	Within 7 days for non-emergency patients, unless benefit of diagnostic capability over risk of acute kidney injury, renal test not available.
SIRM-SIN-AIOM_2022<br>(Follows ESUR_2018)	It is recommended that baseline eGFR be assessed on creatinine value performed within 7 days in patients with unstable or hospitalized renal function, whereas a 3-month interval is considered correct in other patients.
CAR_2022	Within 7 days for inpatients or emergency patients, unless emergent presentation/ benefit of diagnostic capability over risk of acute kidney injury.
ACR_2023	There is no agreed-upon acceptable maximum interval between baseline renal function assessment and contrast medium administration in at-risk patients. Some accept a 30-day interval in outpatients. It seems prudent to have a shorter interval for inpatients, those with a new risk factor, and those with a heightened risk of renal dysfunction.
**What is the eGFR cutoff for patients at risk of acute kidney injury/ needs referring/ further treatment?**	
ESUR_2018	The risk of PC-AKI in patients with eGFR ⩾ 30 ml/min/1.73m2 after intravenous and intra-arterial CM administration with second-pass renal exposure is very low, but there is conflicting evidence on the risk for intra-arterial CM administration with first-pass renal exposure.<br>Preventive measures are recommended for patients with eGFR < 30 ml/min/1.73m2 before intravenous and intra-arterial CM administration with second-pass renal exposure.<br>Preventive measures are recommended for patients with eGFR < 45 ml/min/1.73m2 if they are in ICU or if they will receive intra-arterial CM administration with first-pass renal exposure.
RANZCR_2018	The risk of intravenous contrast media related acute kidney injury (CI-AKI) is likely to be non-existent for patients with eGFR greater than 45 mL/min/1.73m2. No special precautions are recommended in this group prior to or following intravenous administration of iodinated contrast media.<br>The risk is of intravenous CI-AKI is also very likely to be low or non-existent for patients with eGFR 30 - 45 mL/min/1.73m2. Universal use of periprocedural hydration in this group to prevent the theoretical risk of CI-AKI cannot be recommended but patients with impaired function in this range that is acutely deteriorating rather than stable may benefit from this intervention.<br>In patients with severe renal function impairment (eGFR less than 30 ml/min/1.73m2) or actively deteriorating renal function (acute kidney injury) careful weighing of the risk versus the benefit of iodinated contrast media administration needs to be undertaken. Consideration should be given to periprocedural renal protection using intravenous hydration with 0.9% saline (see relevant section). However, severe renal function impairment should not be regarded as an absolute contraindication to medically indicated iodinated contrast media administration.
JRS-JCS-JSN_2018	We continue to regard CKD (eGFR < 60 mL/ min/1.73 m2) as a risk factor for CIN.<br>It is unlikely that the risk of developing CIN increases in CKD patients (eGFR ⩾ 30 mL/min/1.73 m2) after contrast-enhanced CT. However, even if the eGFR is ⩾ 30 mL/min/1.73 m2, it is important to fully evaluate the risk factors for CIN. On the other hand, when contrast-enhanced CT is performed in CKD patients with an eGFR < 30 mL/min/1.73 m2, it is recommended that the risk of CIN onset be explained and appropriate preventive measures be taken as necessary.
UCSF-USC_2020	Limited evidence suggesting intravenous iodinated contrast material is an independent risk factor for AKI in patients with eGFR ⩾ 30 mL/min/1.73 m2. No hydration required.

	This population of patients (eGFR < 30 mL/min/1.73 m2) has the greatest risk for post-contrast acute kidney injury after administration of intravenous iodinated contrast. Contrast is not recommended unless the patient is on dialysis and anuric, or if contrast is considered diagnostically essential. Weigh the benefits of contrast versus potential harms of kidney injury. Pre-procedural prophylaxis with intravenous volume expansion therapy is recommended.
ACR-NKF_2021	Patients with AKI or eGFR less than 30 mL/min/1.73 m2 (including non-anuric patients undergoing maintenance dialysis) should prompt consideration by the referring professional and radiologist to discuss the risks and benefits of contrast media administration
CSR_2021<br>(follows ESUR_2018)	The recommended eGFR risk threshold for patients undergoing enhanced CT examination is 30 ml/min · 1.73 m2. It is safe to directly perform enhanced examination for patients with eGFR ⩾ 30 mL/min/1.73 m2. For patients with eGFR 30~44 mL/min/1.73 m2 and high-risk factors and eGFR < 30 mL/min/1.73 m2, the use of iodine contrast agents can be considered comprehensively, and the relevant information can be explained to the patient before examination before appropriate use.
CSCP-CPA-CSN_2022<br>(follows ESUR_2018)	Preventive measures are recommended for patients with eGFR < 30 ml/min/1.73m2 before intravenous and intra-arterial CM administration with second-pass renal exposure.
SIRM-SIN-AIOM_2022<br>(follows ESUR_2018)	Preventive measures are recommended for patients with eGFR < 30 ml/min/1.73m2 before intravenous and intra-arterial CM administration with second-pass renal exposure. Preventive measures are recommended for patients with eGFR < 45 ml/min/1.73m2 if they are in ICU or if they will receive intra-arterial CM administration with first-pass renal exposure.
CAR_2022	If eGFR >30 mL/min/1.73 m2 and no signs and symptoms of AKI, then proceed with an indicated contrast imaging study. If eGFR ⩽30 mL/min/1.73 m2, or suspected AKI We recommend an individual patient decision involving the caring team, or patient/patient decision-maker to explain and balance the risks of CA-AKI against the risks and uncertainties of delayed or suboptimal imaging.
ACR_2023	At the current time, there is very little evidence that IV iodinated contrast material is an independent risk factor for AKI in patients with eGFR ⩾30 mL / min/1.73m2. Therefore, if a threshold for CI-AKI risk is used at all, 30 mL / min/1.73m2 seems to be the one with the greatest level of evidence. Any threshold put into practice must be weighed on an individual patient level with the benefits of administering contrast material.
**What kind of contrast media is recommended for high-risk patients?**	
ESUR_2018	There is no difference in PC-AKI risk between IOCM and LOCM. The use of ionic, high-osmolar CM should be avoided.
RANZCR_2018	No recommendation.
JRS-JCS-JSN_2018	The risk of developing CIN does not differ between iso- and low-osmolar contrast media. Although there has been no definitive conclusion as to whether the risk of developing CIN differs among the different types of low-osmolar contrast media, there has been no significant difference in the incidence of CIN among them.
UCSF-USC_2020	No recommendation.
ACR-NKF_2021	There are no confirmed clinically relevant differences in risk of CA-AKI between low-osmolality contrast media (LOCM) and iso-osmolarity contrast media (IOCM) for intravenous applications. High-osmolality iodinated contrast media have higher osmolality than do LOCM and IOCM, but high-osmolality iodinated contrast media has been replaced by LOCM and IOCM for intravenous administration in modern clinical practice.
CSR_2021	The use of ionic, high-osmolar CM is with higher risk of PC-AKI. IOCM and LOCM is recommended.
CSCP-CPA-CSN_2022	The risk of PC-AKI is lower in IOCM and LOCM than ionic, high-osmolar CM. IOCM and LOCM is recommended. The ionic, high-osmolar CM is not recommended.
SIRM-SIN-AIOM_2022	No recommendation.

CAR_2022	We do not recommend preferential use of iso-osmolar ICM for reducing risk of CA-AKI; those decisions should be made based on other factors (e.g., cost and availability).
ACR_2023	LOCM are less nephrotoxic than HOCM in patients with underlying renal insufficiency. LOCM were not shown to be significantly different in patients with normal renal function. There is no clear advantage of IV iso-osmolality iodixanol over IV LOCM with regard to CA-AKI or CI-AKI.
**Is reduced contrast media dosage recommended for high-risk patients?**	
ESUR_2018	Use the lowest dose of contrast medium consistent with a diagnostic result.
RANZCR_2018	No recommendation. There is insufficient data to support the idea that warming the contrast media reduces the number of adverse reactions.
JRS-JCS-JSN_2018	There is a possibility that a reduction in the contrast media volume in contrast-enhanced CT may reduce the risk of developing CIN. For patients with a high CIN risk in particular, it is recommended to utilize the minimum amount of contrast media necessary for diagnostic efficacy.
UCSF-USC_2020	No recommendation.
ACR-NKF_2021	If iodinated contrast media is administered to a patient at risk, then a conventional single diagnostic dose should be used (ie, volume typically used for a single diagnostic dose). Ad hoc contrast media dose reductions as an effort to mitigate risk of CI-AKI should be avoided because this practice may produce a suboptimal or nondiagnostic study.
CSR_2021	While maintaining image quality, the amount of iodine contrast agent used can be moderately reduced to avoid delaying diagnosis due to a significant decrease in image quality caused by the use of iodine contrast agent.
CSCP-CPA-CSN_2022	The dosage of iodine contrast agent is a risk factor for CI - AKI. It is recommended to minimize the dosage of iodine contrast agent while meeting clinical diagnosis and treatment needs.
SIRM-SIN-AIOM_2022	It is recommended to administer the minimum dose sufficient to obtain the diagnostic information
CAR_2022	We discourage reduced dosing of IV contrast administration for CT examinations since that lowers parenchymal enhancement and recommended using the appropriate IV dose for high quality CT imaging in all patients.
ACR_2023	It is not recommended to reduce doses to attempt to mitigate the risk of CI-AKI as this may result in suboptimal or nondiagnostic images. Instead, standard contrast dosing is recommended if the benefits have been deemed to outweigh the risks for intravenous iodinated contrast media administration in high-risk patients for CI-AKI.
**How long is the suitable time interval between scan and repeated scan?**	
ESUR_2018	Repeated CM injections in a short period (48–72 h) should be avoided.
RANZCR_2018	No recommendation.
JRS-JCS-JSN_2018	Since repeated contrast-enhanced CT at short intervals may increase the risk of developing CIN, We do not recommend performing a repeat contrast-enhanced CT within 24–48 h of the first.
UCSF-USC_2020	No recommendation.
ACR-NKF_2021	No recommendation.
CSR_2021	Repeated CM injections within 3 days not recommended.
CSCP-CPA-CSN_2022	The repeated use of iodine contrast agents within a short period of time (48-72 hours) in PCI patients is a risk factor for CI - AKI. When there are no other risk factors present, as long as the gI/eGFR does not exceed 1.0, iodine contrast agents can be repeatedly injected; When there are other risk factors, it is best to repeatedly inject iodine contrast agent at a minimum interval of 48 hours and test renal function before this.
SIRM-SIN-AIOM_2022	No recommendation.
CAR_2022	We do not recommend restricting repeat contrast doses in lower risk patients (eGFR >30, no AKI, IV route) or withholding repeat doses for emergency or inpatients who have life-threatening, or acute presentation of illness. We do recommend avoiding repeated

	contrast exposures within 48 hours for elective procedures if the patients at higher risk of CA-AKI (eGFR ≤30, AKI, intraarterial ICM administration). However, in the face of life-threatening illness, repeat dosing of ICM may be necessary and justified to establish a confident diagnosis and treatment plan.
ACR_2023	We do not believe that there is sufficient evidence to specifically endorse the decision to withhold a repeat contrast medium injection until more than 24 hours have passed since the prior injection, nor to recommend a specific threshold of contrast medium volume beyond which additional contrast media should not be given within a 24-hour period. Therefore, the decision to administer closely spaced contrast-enhanced studies is clinical and subjective, with high- risk patients (e.g., Stage IV and Stage V chronic kidney disease, AKI) treated with greater caution than the general population.
**Is hydration recommended for high-risk patients, and how?**	
ESUR_2018	Preventive hydration should be used to reduce the incidence of PC-AKI in at-risk patients. Intravenous saline and bicarbonate protocols have similar efficacy for hydration. For intravenous and intra-arterial CM administration with second pass renal exposure hydrate the patient with either (a) 3 ml/kg/h bicarbonate 1.4% (or 154 mmol/l solution) for 1 h before CM or (b) 1 ml/kg/h saline 0.9% for 3–4 h before and 4–6 h after CM.<br>Oral hydration as the sole means of prevention is not recommended.<br>In patients with severe heart failure (NYHA grade 3–4) or patients with end-stage renal failure (CKD grade V) preventive IV hydration should be individualized by the clinician responsible for patient care.
RANZCR_2018	The risk is of intravenous CI-AKI is also very likely to be low or non-existent for patients with eGFR 30 - 45 mL/min/1.73m2. Universal use of periprocedural hydration in this group to prevent the theoretical risk of CI-AKI cannot be recommended but patients with impaired function in this range that is acutely deteriorating rather than stable may benefit from this intervention. In patients with severe renal function impairment (eGFR less than 30 ml/min/1.73m2) or actively deteriorating renal function (acute kidney injury). Consideration should be given to periprocedural renal protection using intravenous hydration with 0.9% saline (see relevant section). However, severe renal function impairment should not be regarded as an absolute contraindication to medically indicated iodinated contrast media administration.<br>For patients who are at higher risk of CI- AKI, pre and post procedural 0.9% IV saline is recommended as the first line preventive strategy to mitigate the risk of CI-AKI.
JRS-JCS-JSN_2018	We recommend using physiological saline intravenously before and after contrast-enhanced examination in CKD patients, as they are at high risk of developing CIN.<br>We recommend using isotonic solutions to prevent CIN because isotonic 0.9% sodium chloride (physiological saline) is superior to hypotonic 0.45% sodium chloride in preventing CIN. Sodium bicarbonate-based hydration may decrease the<br>risk of developing CIN. When infusion time is limited, administration of sodium bicarbonate-based hydration is recommended. There is no conclusive evidence that short-term intravenous sodium bicarbonate hydration is as effective as standard intravenous hydration for preventing CIN. Excluding emergency cases with limited infusion time, it is recommended to administer infusion for an extended period of time.<br>There is no sufficient evidence that oral water intake is as effective as intravenous hydration therapy in preventing the development of CIN. We recommend that patients receive hydration therapy or other established preventive measures rather than relying on oral water intake to prevent CIN.
UCSF-USC_2020	In patients with eGFR ≥ 30 mL/min/1.73 m2, no hydration required.<br>In patients with eGFR < 30 mL/min/1.73 m2, Pre-procedural prophylaxis with intravenous volume expansion therapy is recommended. The optimal IV volume expansion protocol is unknown and ideally should be tailored to the patient's volume status and medical conditions. Suggested protocols: Inpatients—0.9% normal saline at 100 mL/h IV beginning 6–12 h prior to contrast administration and continuing 4–12 h afterwards; Outpatients—

	0.9% normal saline 500 mL IV bolus prior to contrast administration. Consider post-exposure oral hydration (1 cup of water per hour for 8 h) unless medically contraindicated.
ACR-NKF_2021	Prophylaxis is indicated for patients who have AKI or an eGFR less than 30 mL/min/1.73 m2 and are not undergoing maintenance dialysis. Prophylaxis is not indicated for the general population of patients with stable eGFR greater than or equal to 30 mL/min/1.73 m2, for patients undergoing chronic dialysis, or for patients at risk for heart failure. When prophylaxis is indicated, isotonic volume expansion with normal saline is the preferred method. The ideal timing, volume, and rate of volume expansion is uncertain. Typical volume expansion regimens begin 1 hour before and continue 3–12 hours after contrast media administration, with typical doses ranging from fixed (eg, 500 mL before and after) to weight-based volumes (1–3 mL/kg per hour). Longer regimens (approximately 12 hours) have been shown to lower the risk of CA-AKI compared with shorter regimens. However, longer intravenous protocols are generally impractical in the outpatient setting. Although bicarbonate is likely similar to normal saline for the prevention of CA-AKI, it is not preferred because bicarbonate solutions require pharmacist compounding.<br>Oral hydration has not been well studied for patients with eGFR less than 30 mL/min/1.73 m2 or AKI.
CSR_2021	Physiological saline hydration is currently widely recommended as a preventive method for PC-AKI. The more commonly used hydration regimen is to inject physiological saline intravenously for 1-4 hours before the examination begins and continue for 3-12 hours after the examination. The commonly used infusion volume is 500 ml fixed infusion before and after the use of iodine contrast agent, or a weight adjusted infusion regimen (1-3 ml/kg per hour). A longer infusion duration regimen (approximately 12 hours) can reduce the risk of PC-AKI more effectively than a shorter regimen, but longer hydration regimens are more difficult to implement in outpatient patients. In addition to normal saline, intravenous bicarbonate solution also has a good preventive effect and is not recommended as the first choice.<br>The oral rehydration protocol used in the study was approximately 6 hours before and after the examination. Oral administration of 500-1000 ml of liquid, or hydration at a rate of 1 ml/kg/h for 6-12 hours before and after examination.
CSCP-CPA-CSN_2022	If the patient does not have contraindications for volume expansion, it is recommended to use hydration to prevent CI-AKI.<br>Oral hydration is not recommended as the first or only preventive strategy<br>Both physiological saline and sodium bicarbonate solution can be used as hydration crystal solutions, and suitable hydration crystals can be selected according to clinical needs.<br>The advantages and disadvantages of different hydration schemes are still unclear, and further research is needed on the optimal individualized hydration scheme (such as the speed, volume, and time of intravenous infusion).
SIRM-SIN-AIOM_2022	Hydration/volume expansion represents the gold standard of PC-AKI preventive therapy, keeping in mind that any intervention must be proportionate to the patient’s overall risk;<br>Intravenous hydration is recommended as a preventive measure for patients with moderate risk of PC-AKI (intravenous or intra-arterial administration with second renal passage with GFR < 30) using either Na bicarbonate (NaBic) 1.4% 3 ml/Kg/hr for 1 h prior to administration, or saline 1 ml/Kg/hr for 3–4 h before and 4–6 h after administration.<br>Two meta-analyses by Meier show that NaBic is more useful than saline when there is no time to perform prolonged hydration and thus may be more appropriate in emergency procedures<br>The prevention of PC-AKI in patients at very high risk (due to comorbidity and to the procedure itself) undergoing procedures with intra-arterial administration with first renal passage remains debated, and for this reason We recommend that each center should have an individualized prevention protocol for this type of patients
CAR_2022	We do not recommend oral or intravenous hydration for patients with eGFR >30 mL/min/1.73m2, receiving intravenous or intra-arterial ICM.

	For patients with eGFR ≤30 mL/min/1.73m2, receiving intravenous ICM, there is a lack of evidence on benefit of volume expansion. Hence the working group makes no recommendation in this regard; institutions may choose practices best suited to their local environments.
ACR_2023	The major preventive action to mitigate the risk of CI-AKI is to provide intravenous volume expansion prior to contrast medium administration. Isotonic fluid such as 0.9% normal saline (NS) is preferred. Typical prophylaxis regiments begin 1 hour prior to the exam and continue 3-12 hours after with longer regiments (approximately 12 hours) shown to lower the risk of CA-AKI compared with shorter regiments. Typical doses may be fixed volume (e.g., 500 mL NS) before and after or weight- based volumes (1-3mL/kg per hour). Oral hydration has not been well studied for patients with eGFR less than 30 mL/min/1.73 m2 or in patients with AKI. Bicarbonate is likely similar to normal saline for the prevention of CA-AKI, but it is not preferred due to the additional requirement for pharmacist compounding.
**Is any drug recommended for high-risk patients, and what are they?**	
ESUR_2018	Not recommended: N-Acetylcysteine; statins; ACE inhibitors or angiotensin receptor blockers; Vitamin C
RANZCR_2018	Not recommended: N-Acetylcysteine
JRS-JCS-JSN_2018	Not recommended: N-Acetylcysteine; human atrial natriuretic peptide (hANP); Vitamin C; statins
UCSF-USC_2020	No recommendation.
ACR-NKF_2021	Not recommended: N-Acetylcysteine
CSR_2021	Not recommended: N-Acetylcysteine; statins; ACE inhibitors or angiotensin receptor blockers; Vitamin C
CSCP-CPA-CSN_2022	Not recommended: N-Acetylcysteine; RAAS inhibitor; Vitamin C; statins; alprostadil; trimetazidine; theophylline; human atrial natriuretic peptide (hANP)
SIRM-SIN-AIOM_2022	Not recommended: N-Acetylcysteine; statins
CAR_2022	Not recommended: N-acetylcysteine; statins; theophylline; prostaglandin E1; nicorandil; Vitamin C; allopurinol; alpha-tocopherol; fenoldopam; natriuretic peptides; trimetazidine
ACR_2023	Not recommended: N-Acetylcysteine; mannitol; furosemide; theophylline; endothelin-1; fenoldopam
**Is blood purification therapy recommended for high-risk patients, and how?**	
ESUR_2018	Renal replacement therapy has not been shown conclusively to reduce the risk of PC-AKI in patients receiving intravenous or intra-arterial CM, and its use is NOT recommended.
RANZCR_2018	No recommendation.
JRS-JCS-JSN_2018	Blood purification therapy after administering contrast media does not decrease the risk of developing CIN and is not recommended. In particular, hemodialysis therapy is not recommended.
UCSF-USC_2020	HD patients who receive IV contrast do not require routine post-procedural dialysis.
ACR-NKF_2021	Because of the inherent demonstrated lack of benefit, risks, and cost, neither acute dialysis nor continuous renal replacement therapy should be initiated or have the schedule changed solely based on iodinated contrast media administration, regardless of residual kidney function
CSR_2021	Intravenous iodine contrast agents can be used for patients without renal function undergoing dialysis, and emergency dialysis is not recommended. However, the use of iodine contrast agents can be synchronized with scheduled hemodialysis or hemofiltration. It is not recommended to use emergency kidney replacement therapy to remove iodine contrast agents, but it can be used as appropriate when excessive iodine contrast agents are given, there is obvious cardiac dysfunction or electrolyte or acid-base balance abnormalities that significantly worsen the patient's general condition.
CSCP-CPA-CSN_2022	It is not recommended to routinely use dialysis for CI - AKI, and dialysis treatment can only be considered when the condition is seriously life-threatening and there are indications for dialysis.

SIRM-SIN-AIOM_2022	It is not recommended to coordinate dialysis after iodine- based contrast agent infusion or to provide an additional dialysis session to remove it.
CAR_2022	We do not recommend any form of post ICM administration renal replacement therapy, either dialysis or continuous renal replacement therapy for reduction of the risk of CA-AKI.
ACR_2023	Patients should not have acute dialysis nor continuous renal replacement therapy initiated or alter their schedule solely based on iodinated contrast media administration regardless of renal function due to the risks, costs and lack of benefit.

From:

[1] European Society of Urogenital Radiology (2018) ESUR Guidelines on contrast agents. Available via https://www.esur.org/wp-content/uploads/2022/03/ESUR-Guidelines-10_0-Final-Version.pdf Accessed 15 July 2023.

[2] The Royal Australian and New Zealand College of Radiologists (2018) Iodinated contrast media guideline, version 2.3. Available via https://www.ranzcr.com/search/ranzcr-iodinated-contrast-guidelines Accessed 15 July 2023.

[3] Isaka Y, Hayashi H, Aonuma K et al; Japanese Society of Nephrology, Japan Radiological Society, Japanese Circulation Society Joint Working Group (2020) Guideline on the use of iodinated contrast media in patients with kidney disease 2018. Jpn J Radiol 38(1):3-46

[4] Huynh K, Baghdanian AH, Baghdanian AA, Sun DS, Kolli KP, Zagoria RJ (2020) Updated guidelines for intravenous contrast use for CT and MRI. Emerg Radiol 27(2):115-126

[5] Davenport MS, Perazella MA, Yee J et al (2020) Use of intravenous iodinated contrast media in patients with kidney disease: consensus statements from the American College of Radiology and the National Kidney Foundation. Radiology 294(3):660-668

[6] Quality Control and Safety Management Committee of Chinese Society of Radiology Chinese Medical Association (2021) Expert consensus of iodinated contrast agent use in patients with renal diseases. Chin J Radiol 55(6):580-590 [Article in Chinese]

[7] Chinese Society of Clinical Pharmacy, Hospital Pharmacy Professional Committee of Chinese Pharmaceutical Association, Chinese Society of Nephrology (2022) Expert consensus on prevention and treatment of iodine contrast media-induced acute kidney injury. Chin J Nephrol 38(3):265-288 [Article in Chinese]

[8] Orlacchio A, Guastoni C, Beretta GD et al (2022) SIRM-SIN-AIOM: appropriateness criteria for evaluation and prevention of renal damage in the patient undergoing contrast medium examinations-consensus statements from Italian College of Radiology (SIRM), Italian College of Nephrology (SIN) and Italian Association of Medical Oncology (AIOM). Radiol Med 127(5):534-542

[9] Macdonald DB, Hurrell C, Costa AF et al (2022) Canadian Association of Radiologists guidance on contrast associated acute kidney injury. Can Assoc Radiol J 73(3):499-514

[10] American College of Radiology (2023) ACR manual on contrast media. Available via https://www.acr.org/Clinical-Resources/Contrast-Manual Accessed 15 July 2023

Supplementary Table S6 Formula for eGFR calculation

**Population**	**Equation**
CKD-EPI equation (serum creatinine, sCr in µmol/L; age in years. All equations × 1.159 if African American race) [1]	
Female sCr ⩽ 62 µmol/L	$144 \times (sCr / 62)^{-0.329} \times 0.993^{Age}$
Female sCr > 62 µmol/L	$144 \times (sCr / 62)^{-1.209} \times 0.993^{Age}$
Male sCr ⩽ 80 µmol/L	$141 \times (sCr / 80)^{-0.411} \times 0.993^{Age}$
Male sCr > 80 µmol/L	$141 \times (sCr / 80)^{-1.209} \times 0.993^{Age}$
Revised Schwartz equation (sCr in µmol/L; patient length in cm) [2]	
Children	36.5 × Length / sCr

From:

[1] Delanaye P, Ebert N (2012) Assessment of kidney function: estimating GFR in children. Nat Rev Nephrol 8(9):503-504

[2] Schwartz GJ, Muñoz A, Schneider MF et al (2009) New equations to estimate GFR in children with CKD. J Am Soc Nephrol 20(3):629-637
